# Stereoisomeric Effects of Diammoniumcyclohexane Counterions on the Self-Assembly of Amino Acid-Based Surfactants

**DOI:** 10.3390/molecules30204114

**Published:** 2025-10-16

**Authors:** Saylor E. Blanco, Nathan Black, Margarita A. Alvarez, Kevin F. Morris, Mark A. Olson, Eugene J. Billiot, Fereshteh H. Billiot

**Affiliations:** 1 Department of Physical and Environmental Sciences, Texas A&M University Corpus Christi, 6300 Ocean Dr., Corpus Christi, TX 78412, USA; sblanco3@islander.tamucc.edu (S.E.B.); mark.olson@tamucc.edu (M.A.O.);; 2 Department of Chemistry, University of Washington, 4000 15th Avenue NE, Seattle, WA 98195, USA; 3 Department of Chemistry, Carthage College, 2001 Alford Park Drive, Kenosha, WI 53140, USA

**Keywords:** amino acid surfactants, diammonium counterions, critical micelle concentration, self-assembly, *cis*-*trans* counterion stereochemistry, biodegradable surfactants

## Abstract

The impact of counterion structure, especially variations in constitutional and stereochemical isomers, on the properties and performance of AABSs remains under-explored. This study investigates how structural variations, particularly the stereochemistry of diammonium cyclohexane (DACH) counterions, influence the self-assembly behavior of AABSs. Four AABSs: undecanoyl-glycine, -L-alanine, -L-valine, and -L-leucine, were paired with six DACH counterions representing *cis*/*trans* isomers of 1,2-, 1,3-, and 1,4-DACH. Critical micelle concentrations (CMCs) were determined via conductimetry, and micellar sizes were measured using dynamic light scattering. The degree of counterion binding (β) was calculated to probe micelle stability, while geometry-optimized structures of the DACH isomers were obtained using density functional theory. Lastly, pH measurements were taken to probe the protonation of DACH counterions at their natural pH, where both the DACH counterion and AABS headgroups intrinsically behave as buffers. Results indicate that while surfactant hydrophobicity primarily dictates CMC in other AABS/DACH combinations, *trans*-1,3-DACH leads to consistently higher CMCs. This deviation likely arises from its structural conformation, which positions the amine groups an intermediate distance of ~4.4–4.5 Å apart, allowing a small fraction of divalently charged counterions to form strong electrostatic bridging pockets at the micelle interface. These interactions dominate over headgroup effects, leading to elevated and surfactant-independent CMC values. Regarding size and other unusual trends in the systems, *cis*- isomers formed slightly larger micelles, and *trans*-1,4-DACH induces abnormal aggregation in undecanoyl-glycine leading to temperature dependent gel formation. These findings highlight the significant influence of counterion structure on AABS behavior and support counterion design as a strategy for enhancing surfactant performance in sustainable applications.

## 1. Introduction

Traditional non-biodegradable surfactants, widely used in household, industrial, and agricultural applications, such as branched alkylbenzene sulfonates [1], alkylphenol ethoxylates [2], quaternary ammoniums [3,4], and perfluoroalkyl surfactants [5], pose significant environmental risks due to their persistence in aquatic systems and potential toxicity to wildlife [6]. The demand for eco-friendly alternatives has driven interest in biodegradable surfactants derived from renewable resources. Amino acid-based surfactants (AABSs) offer a promising solution, as they are derived from amino acids and fatty acids, and thus exhibit biodegradability, low toxicity, and structural diversity [6,7,8,9,10,11,12,13]. Moreover, AABSs have a wide range of applications, including drug delivery, antibacterial formulations, cosmetics, agriculture, and household cleaning products [6,7,8,9,10]. Due to their ability to facilitate mixing of oil and aqueous phases, they are also used in material science and petroleum oil recovery, while maintaining environmental efficacy [8,11]. Their amphiphilic properties can be precisely tuned by modifying the amino acid type, hydrophobic tail, or counterion, allowing for the design of surfactants tailored to specific applications. This tunability is critical for optimizing performance while minimizing environmental impact. However, there has been limited systematic exploration into how structural modifications, particularly those generating constitutional and stereochemical isomers of divalent counterions, affect the properties and behaviors of AABSs.

A principal characteristic of a surfactant’s aggregation capability is its critical micelle concentration (CMC), which can be significantly influenced by alterations to the surfactant’s counterions. Micellar systems with low CMC values are commonly utilized in detergents and cleaning foams, where a low CMC enables greater dilution without sacrificing efficiency [14]. Surfactants can also self-assemble into bilayers forming vesicles with hollow interiors that can be used for drug delivery systems, and those with ultralow CMCs can effectively encapsulate hydrophobic drugs, enhancing their solubility [15,16]. The choice of counterion impacts many of these parameters, including micellar size, which is critical for applications requiring either precise targeting (small micelles) or high drug loading capacity (larger aggregates) [17,18,19,20]. Additionally, counterion properties such as size, steric bulk, charge, and spacer length between charged groups significantly affect the degree of counterion binding and, consequently, the stability of micellar structures. Studies indicate that factors like spacer length and *cis*-*trans* isomerization in counterion molecules notably influence micellar characteristics, including the formation of counterion binding pockets [13,21,22,23,24,25,26,27]. For instance, in micelles formed by alkyl and tetraalkyl ammonium sulfates, increasing monovalent counterion size from Li^+^ to Cs^+^ reduces electrostatic repulsion and enhances screening, thereby lowering the CMC and producing larger aggregates [28,29]. Thus, the tuning of surfactant properties by modifying counterions represents a crucial strategy in optimizing micelle-based systems.

**Figure 1:**
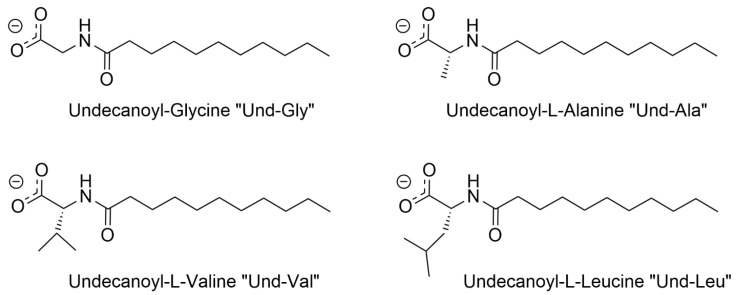
Structural representations of the carboxylate anions of the AABSs utilized in the study with their respective abbreviations.

**Figure 2:**
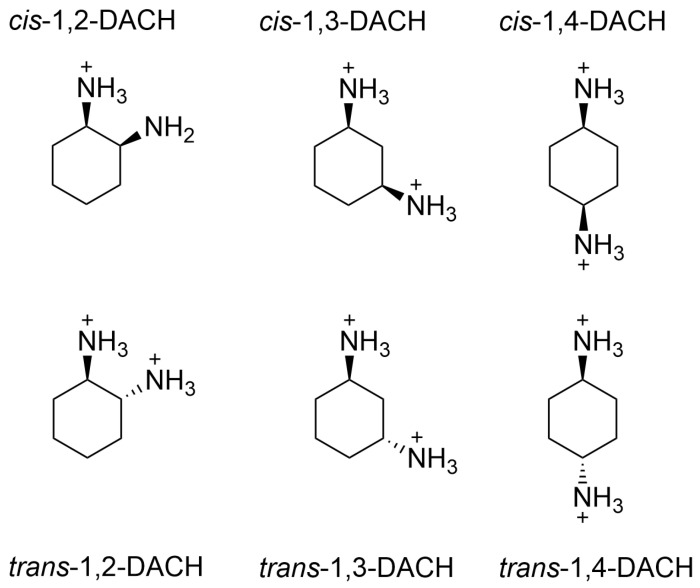
Structural representations of the *cis*- and *trans*- stereoisomers of 1,2-DACH, 1,3-DACH, and 1,4-DACH counterions. Fully protonated counterions are shown; however, the exact diamine charge will depend on the pH.

This research aims to characterize four AABSs (Figure 1): undecanoyl-glycine (Und-Gly), undecanoyl-L-alanine (Und-Ala), undecanoyl-L-valine (Und-Val), and undecanoyl-L-leucine (Und-Leu) in the presence of six diammonium cyclohexane (DACH) counterions: (*cis*/*trans*)-1,2-DACH, (*cis*/*trans)*-1,3-DACH, and (*cis*/*trans*)-1,4-DACH (Figure 2). The use of cyclic isomers of diamine cyclohexane as counterions in amino acid–based surfactants has not been previously explored, particularly in relation to their effect on micellization. This manuscript is the first to describe these interactions, highlighting their importance in understanding how counterion structure influences surfactant self-assembly. The counterions are cyclohexane diamine and they differ in the location of the two amines on the ring. The AABSs differ in side chain length of their amino acid headgroup. The counterions were chosen as an extension of a previous study that investigated the micelle-binding of linear diammonium counterions bearing alkyl chains of differing lengths bridging the ammonium centers [21]. Here the six cyclic counterions are constitutional isomers differing in the position of their diammonium centers relative to each other in the 1,2-, 1,3-, and 1,4- positions with each existing as either a *cis*- or *trans*- diammonium stereoisomer. The CMC of each AABS and counterion combination was measured by variable concentration conductimetry in natural pH, which is reported for each system along with the percent protonation of the counterions since these mixtures inherently act as buffers. From the conductimetry data, the degree of counterion binding (β) for each system was also calculated. Micellar size was measured using photon correlation spectroscopy/dynamic light scattering (DLS). Finally, density functional theory (DFT) was employed to calculate energy-minimized structures of the DACH counterions at two different protonation states.

## 2. Results and Discussion

### 2.1. Structural Effects on Critical Micelle Concentration

**Figure 3:**
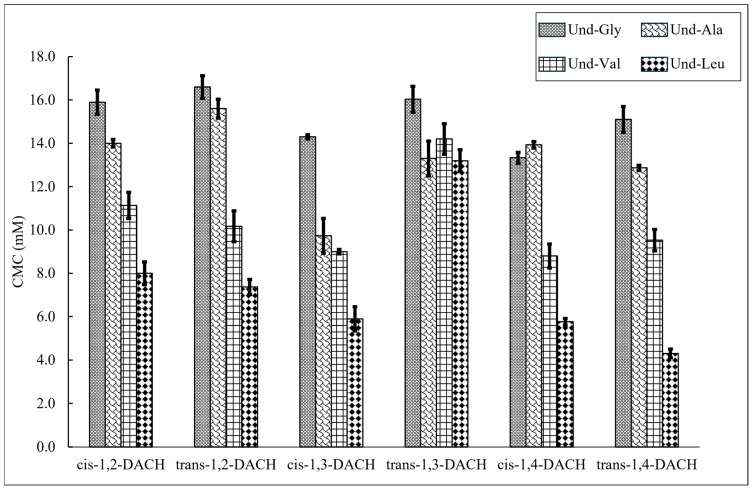
CMC values measured by variable concentration conductivity experiments for each AABS coupled with their respective DACH counterions. Data for Und-Gly with the *trans*-1,4-DACH counterion is not shown as it was insoluble at room temperature.

CMC values were determined by conductimetry for surfactants Und-Gly, Und-Ala, Und-Val, and Und-Leu with each of the counterions, *cis*-1,2-DACH, *trans*-1,2-DACH, *cis*-1,3-DACH, *trans*-1,3-DACH, *cis*-1,4-DACH and *trans*-1,4-DACH (Figure 3, Table 1). It was determined that the CMCs of the surfactants decrease with increases in the length of the amino acid side chain in the presence of most counterions. For example, for solutions containing *cis*-1,2-DACH, the CMC steadily decreased from 15.9 mM to 8.0 mM as the amino acid side chain length increased from Und-Gly to Und-Leu. This observation is likely due to the increased hydrophobic character associated with the larger amino acid side chains, which would promote micellization at lower concentrations to reduce unfavorable hydration interactions. Interestingly, this trend is not followed by the surfactant systems comprising *trans*-1,3-DACH. Rather, the CMCs for these systems ranged from 13.2 mM to 16.0 mM, with most values being statistically indistinguishable. This result suggests that *trans*-1,3-DACH binds to the micelles in such a way as to minimize the effect of varying headgroup hydrophobicity of each surfactant during micelle formation. Additionally, the CMC of Und-Gly in the presence of *trans*-1,4-DACH could not be measured at 25 °C due to its poor solubility and gel formation. Instead, a CMC of 15.1 mM was obtained, albeit at 60 °C. The insolubility of this system at 25 °C indicated that 1,4-DACH binds to Und-Gly in a way that promotes the self-assembly of larger aggregate morphologies at low temperatures. These assemblies can subsequently be disrupted at elevated temperatures. Overall, the data indicated that the structural parameters that influence CMC appear to be primarily driven by differences in the AABS side chains rather than counterion structure, with exceptions exhibited by both *trans*-1,3-DACH and *trans*-1,4-DACH.

AABS CMCs are also selectively influenced by the stereochemistry of the DACH counterions. For example, the stereochemical identity, either *cis-* or *trans-*, of the 1,3-DACH counterions, greatly affects the CMC of their corresponding AABSs. In the case of Und-Leu, its CMC is 5.9 mM while accompanied by the *cis*-1,3-DACH counterion and 13.2 mM with the *trans*-1,3-DACH counterion. This result indicates that the relative spatial arrangement of the cationic 1,3-DACH ammonium centers, as determined by their stereochemistry, has a profound effect on AABS aggregation behavior. However, this is not true for both the 1,2-DACH and 1,4-DACH stereoisomers. In the case of 1,2-DACH stereoisomers, the CMCs of most AABSs are statistically indistinguishable. For example, CMCs of 8.0 ± 0.5 mM and 7.4 ± 0.4 mM were measured for Und-Leu in the presence of *cis*-1,2-DACH and *trans*-1,2-DACH, respectively. It is likely that *trans*-1,3-DACH counterions have a different mechanism of micelle-binding, leading to CMCs that are largely independent of AABS side chain structure as discussed earlier.

Upon further analysis, the stereochemical influence of the DACH counterions on the CMC of the AABSs stems directly from the relative positions, axial (*a*) versus equatorial (*e*), of their diammonium centers that are inherent to the 1,2-, 1,3-, and 1,4-disubstituted cyclohexane structure as it adopts a chair conformation as either the *cis*- or *trans*- stereoisomer (Table 1). For example, *cis*-1,2-DACH is a C_1_ symmetric meso compound that is optically inactive and structurally confined to existing as an axial-equatorial (*ae*) conformer, with the positioning of one ammonium center at an axial position and the other ammonium center at an equatorial position. Chair inversion of *cis*-1,2-DACH results in yet another *ae* conformer. *Trans*-1,2-DACH, on the other hand, possesses C_2_ symmetry, is optically active, and can exist as both an *aa* or an *ee* conformer, but is energetically relegated to adopting an *ee* conformation in about 99:1 excess of *ee* to *aa* [30]. In a similar fashion to 1,2-DACH, *cis*-1,4-DACH is also a meso compound that is optically inactive and structurally confined to existing as an *ae* conformer, for which chair inversion results in yet another *ae* conformation. *Trans*-1,4-DACH is a C_2h_ optically inactive compound, that in the same manner as *trans*-1,2-DACH, is forced to exist as an *ee* conformer, whereby the chair inverted *aa* conformation is energetically unfavorable. The stereochemical similarities between 1,2- and 1,4-DACH is clear, whereby the *cis*- isomers for both 1,2- and 1,4-DACH exists as *ae* conformers, and the *trans* isomers for both 1,2- and 1,4-DACH exists as *ee* conformers. These stereochemical similarities a priori, account for the similar manner with which the *cis*- and *trans*- stereoisomers for both the 1,2-DACH and 1,4-DACH counterions interact with the AABSs, as previously mentioned. It should be noted that the exact conformation of the DACH counterions in solution may vary; however, the preference for each counterion with their respective *aa*, *ee*, and *ae* geometry is consistent.

The *cis*- and *trans*- structures of 1,3-DACH are stereochemical opposites with regard to *a* and *e* conformers as compared to 1,2- and 1,4-DACH [30,31]. *Cis*-1,3-DACH is a C_S_ symmetric meso compound that is optically inactive and exists exclusively as an *ee* conformer, for which the chair-inverted *aa* conformer is highly energetically unfavorable on account of 1,3-diaxial strain. *Trans*-1,3-DACH is C_1_ symmetric, optically active, and structurally confined to existing as an *ae* conformer. Chair inversion of *trans*-1,3-DACH simply results in yet another *ae* conformer. As a result, both *cis*- and *trans*- isomers of 1,3-DACH interact with the AABSs in a fundamentally different manner than both the 1,2- and 1,4-DACH counterions.

### 2.2. Factors Affecting Micellar Hydrodynamic Diameter

To investigate the effect of the DACH counterions on the self-assembly of AABS into micellar aggregate structures, especially those with abnormal CMC measurements (*trans*-1,3-DACH and *trans*-1,4-DACH), the hydrodynamic diameters (H_D_) of the micelles formed in solution were measured using DLS (Table 2). For most AABS-counterion pairs investigated, there is a modest increase in micellar size as the complexity of the AABS headgroup increases from Und-Gly to the Und-Leu. For example, the H_D_ of systems containing the *cis*-1,3-DACH counterion gradually rises from Und-Gly (2.0 nm) to Und-Leu (2.7 nm). The extended side chain structures associated with larger AABSs likely lead to minor steric repulsions between surfactant headgroups, causing the formation of slightly larger aggregates. This trend demonstrates that while AABS aggregation is largely governed by surfactant hydrophobicity, steric repulsions play a minor role in the self-assembly process.

Micellar H_D_ also exhibits a dependence on the stereochemical identity of the DACH counterions. Namely, AABS micelles tend to be slightly larger when formed in the presence of a *cis*-1,2- and *cis*-1,3-DACH counterion compared to that of its *trans*- counterpart. Und-Gly exhibits H_D_ sizes of 2.3 and 1.7 nm in the presence of *cis*-1,2-DACH and *trans*-1,2-DACH, respectively. This difference is likely due to the altered spatial arrangements of the *cis*- versus the *trans*- counterions as they are situated and bound at the micellar surface. According to DFT calculations, which will be discussed in more depth in the next section, the inter-nitrogen distance for *cis*-1,2-DACH is 2.75 Å as compared to 2.73 Å for *trans*-1,2-DACH, at a pH where both stereoisomers predominantly exist in the +1 charged state. When bonding monovalently, the distance between the two amines is less relevant for variability of size than it is for DACH counterions with more linear protrusions. Moreover, the distance from the charged ammonium center to the furthest atom in the opposite direction on the molecule is 5.33 Å and 5.30 Å for *cis*- and *trans*-1,2-DACH counterions, respectively. Despite the odd behavior leading to consistent CMC values, there is no major exclusion of systems containing *trans*-1,3-DACH counterions from the other systems. The perpendicular, spatially extended binding configuration proposed here is consistent with the broader micellar expansion observed in systems incorporating *trans*-DACH counterions, particularly for the 1,3- and 1,4-substituted isomers, which adopt more linear or sterically extended geometries.

**Figure 4:**
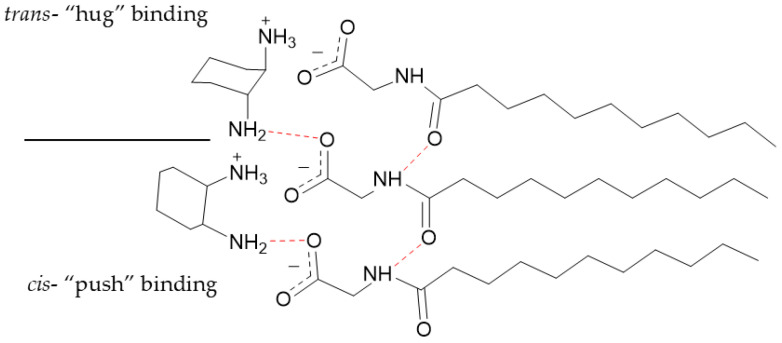
Graphical representation of the proposed binding of *cis-* and *trans-* DACH counterions to a micellar surface upon counterion condensation. Red dashed lines depict hydrogen bonding. Partially protonated counterions are shown; however, the exact diamine charge will depend on the pH.

These minor differences between inter-nitrogen length however are likely not significant enough to cause size differences between *cis*- and *trans*- iteration of 1,2-DACH. This difference as well as size differences between the other *cis*- and *trans*- steroisomers is likely due to the altered spatial arrangements of the *cis*- versus the *trans-* counterions as they are situated and bound at the micellar surface. More specifically, both ammonium centers of the *cis*-DACH counterion are oriented in the same relative direction with respect to the larger cyclohexane core. This arrangement optimizes ion-pairing during counterion condensation, allowing for the cationic *cis*-DACH ammonium center to bind to the anionic micellar surface in a way that positions their cyclohexane cores outward from the charged micelle. While one ammonium center is binding electrostatically, the neutral one forms a hydrogen bond with oxygen on the carboxylate group of the adjacent surfactant. From the perspective of the cyclohexane cores, this behavior can be described as “pushing” the ammonium centers toward the micellar surface. This behavior is consistent with our pH data, which indicates that most counterions exist predominantly in the +1 state rather than as fully +2 species. The *trans*-DACH counterions, however, orient their ammonium centers in opposing directions, causing the cyclohexane core and therefore the counterion to bind closer to the AABS headgroups. In this behavior, the cyclohexane cores can be described as “hugging” the micellar surface with their ammonium centers. The proposed “push” and “hug” binding modes of the DACH stereoisomers are depicted in Figure 4.

This trend is not present for AABSs in the presence of 1,4-DACH counterions. For these systems, the measured H_D_ values for AABSs under the influence of *trans* counterions are larger than their *cis* counterparts. Und-Leu exhibits H_D_ sizes of 3.1 and 2.7 nm in the presence of *trans*-1,4-DACH and *cis*-1,4-DACH, respectively. This result is likely caused by the orthogonal positioning and relatively large distances between the cationic ammonium centers of the *trans*-1,4-DACH counterions, affectively hindering adoption of the “hug” binding mode. Rather, it is likely that only one of *trans*-1,4-DACH’s ammonium centers binds to the micellar interface, while the other protrudes perpendicularly. Due to the opposite positioning of the ammonium centers on the cyclohexane core, it is likely that *cis*-1,4-DACH also binds in this monovalent orientation. This monovalent or perpendicular model of 1,4-DACH binding to AABS was also proposed by Fletcher et al. in an NMR study of counterion binding to undecylvaline leucinate micelles [23]. Altogether, these altered binding behaviors may explain the reversal in micellar H_D_ sizes induced by stereochemical identity of the 1,4-DACH counterions. This deviation of *trans*-1,4-DACH from the proposed “push” and “hug” binding modes can also be used to rationalize its abnormally low solubility (and associated binding interactions) with Und-Gly, as noted earlier.

To specifically probe the temperature-dependent morphologies of Und-Gly in the presence of *trans*-1,4-DACH, H_D_ measurements were made as a function of temperature, from 75 °C to 10 °C. Starting at 75 °C, the micelle H_D_ size remained stable at ~4.3 nm until the temperature reached 45 °C. Upon reaching 45 °C, the micellar H_D_ increased, ranging from 312.1 to 338.7 nm at and below 55 °C. The observed insolubility at lower temperatures was likely due to the formation of these larger micellar morphologies, which then go on to fall out of solution.

**Figure 5:**
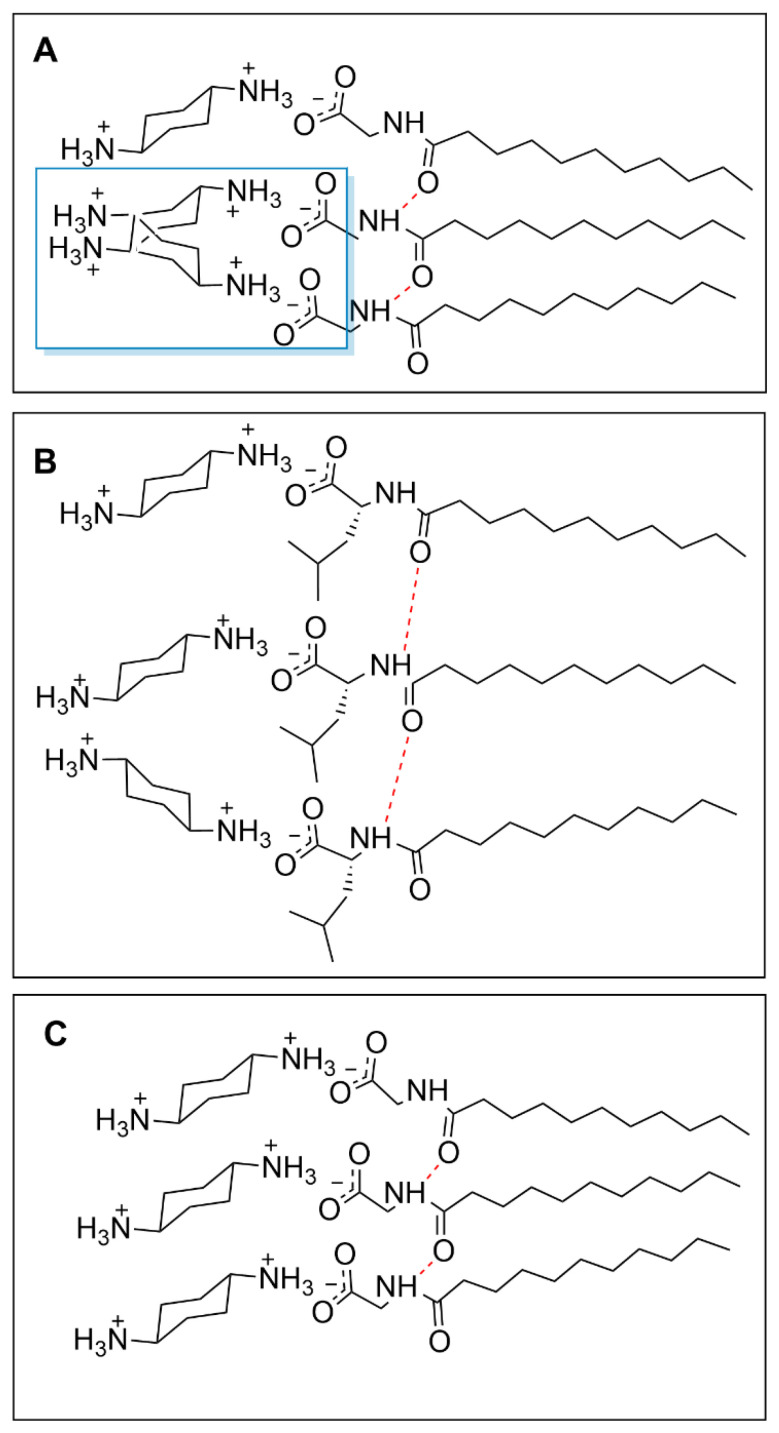
Structural representations of the proposed binding schema of Und-Gly with *trans*-1,4-DACH (**A**) the boxed region shows binding interference between counterions on adjacent surfactants, compared to Und-Leu with *trans*-1,4-DACH (**B**), and the organized alignment of Und-Gly with *trans*-1,4-DACH (**C**) leading to gel formation. Fully protonated counterions are shown; however, the exact diamine charge will depend on the pH.

Even when completely soluble at higher temperatures, the micelle H_D_ size (4.3 nm) is much larger than the other micellar aggregates. The observation of these relatively large aggregates, along with dramatic changes in micellar H_D_ with changes in temperature, support the conclusion that the interactions between Und-Gly and *trans*-1,4-DACH are different from those present in the other AABS-counterion pairs investigated. This difference can be explained by Und-Gly’s small side chain, which enables strong hydrogen bonding between the amide function groups of contiguously assembled AABS molecules, leaving little space between adjacent headgroups. This crowding of Und-Gly’s headgroups hinders the ability of *trans*-1,4-DACH counterions to bind to the micellar interface. Furthermore, because *trans*-1,4-DACH’s predicted binding mode has only one ammonium center in contact with the anionic carboxylate site of the Und-Gly headgroup, its reduced charge-stabilization (compared to other DACH counterions) is likely unable to overcome the strong hydrogen bonding between the Und-Gly headgroups as depicted in Figure 5A. This does not present an issue for AABSs with increased headgroup separation leading to reduced hydrogen bonding strength and adequate room for counterion bonding as depicted in Figure 5B. The issue of steric hindrance between neighboring counterions is minimal for more compact or bent isomers due to their reduced exposure and lower interfacial charge density, despite perpendicular bonding.

The tight lamellar network of adjacent surfactants in Und-Gly/*trans*-1,4-DACH systems depicted in Figure 5C would therefore explain the insoluble gel formations that were observed at lower temperatures. At higher temperatures, increased thermal energy likely facilitates a more favorable alignment of the counterions by disrupting the amide hydrogen bonds in Und-Gly. The reduced repulsion allows the surfactants to pack more tightly and form elongated micelles as previously explained, giving rise to the increased size relative to other systems (4.3 nm) which is further emphasized upon the formation of a gel when cooling. The observed deviations in size therefore reflect the system’s unique structural characteristics rather than temperature effects alone. This phenomenon of temperature-dependent gel formation has been observed in a variety of surfactant systems [32,33].

### 2.3. Degree of Counterion Binding

**Figure 6:**
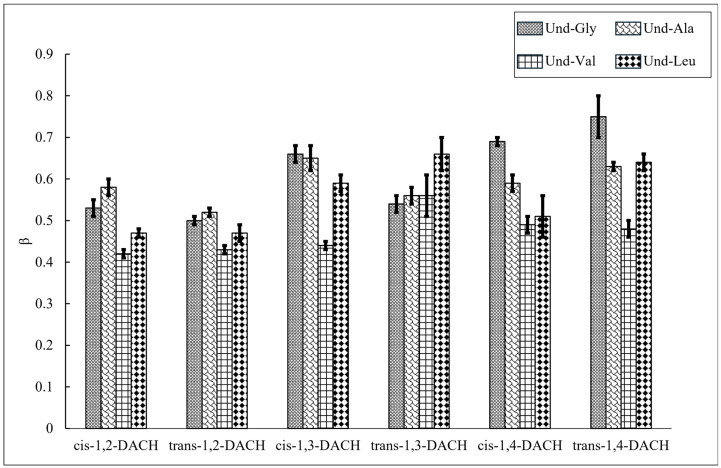
The degree of counterion binding (β) of each AABS with respect to DACH counterions.

To investigate the efficacy of counterion condensation upon micelle formation, the degree of counterion binding (β) was calculated from conductimetry according to Equation (1) (Section 3.6) for each AABS and counterion pair (Figure 6, Table 3). For most DACH counterions, Und-Val consistently exhibited lower β values than the other AABSs. This observation is most pronounced when Und-Val was ion-paired with *cis*-1,3-DACH, for which β = 0.44, corresponding to 44% binding. AABSs Und-Ala and Und-Leu (Und-Val’s structural neighbors) exhibited 65% and 59% binding, respectively. This result indicates that the steric bulk presented by Und-Val’s amino acid side chain hinders DACH counterion binding in a manner that AABSs with similar side chains do not. Despite its steric bulk, Und-Leu does not exhibit a low degree of counterion binding. Its longer side chain leads to steric repulsion between AABS headgroups, giving the counterions greater access to the anionic carboxylate groups, thereby augmenting counterion binding. This selective reduction in Und-Val’s counterion binding (compared to Und-Ala and Und-Leu) agrees with previous findings, albeit with AABSs coupled with linear diammonium counterions [21].

AABS-*trans*-1,3-DACH salts undergo aggregation behavior that does not follow this general trend, as it exhibits an identical degree of micellar binding (56%) for both Und-Ala and Und-Val. In addition, the other β values measured for the *trans*-1,3-DACH counterion paired AABSs were not statistically different, indicating consistent binding behavior for the *trans*-1,3-DACH counterion regardless of the structure of the AABS side chain. This result can be explained by the inherent *ae* conformer structure of the *trans*-1,3-DACH counterion, which can accommodate a range of side chain lengths, from Und-Gly to Und-Leu, in interstitial space formed when bound and ion-paired to the micellar superstructure. This interstitial space may partially insulate AABS side chains from unfavorable hydration contacts, reducing the thermodynamic drive towards micellization.

**Figure 7:**
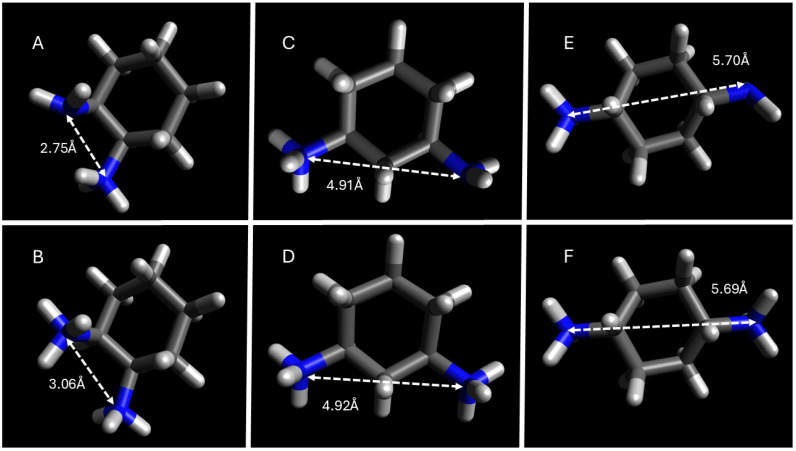
DFT geometry optimization of DACH counterions with the distance in angstroms between amines. (**A**). *cis*-1,2-DACH, (**C**). *cis*-1,3-DACH, (**E**). *cis*-1,4-DACH, all exhibiting a +1 charge, and (**B**). *cis*-1,2-DACH, (**D**). *cis*-1,3-DACH, and (**F**). *cis*-1,4-DACH all exhibiting a +2 charge.

**Figure 8:**
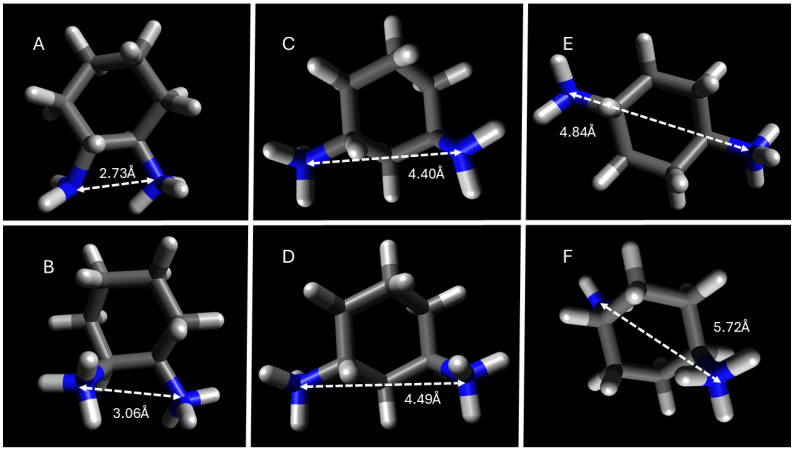
DFT geometry optimization of DACH counterions with the distance in angstroms between amines. (**A**). *trans*-1,2-DACH, (**C**). *trans*-1,3-DACH, (**E**). *trans*-1,4-DACH, all exhibiting a +1 charge, and (**B**). *trans*-1,2-DACH, (**D**). *trans*-1,3-DACH, and (**F**). *trans*-1,4-DACH all exhibiting a +2 charge.

In addition to its more favorable solubility in aqueous solutions, this dampening of the hydrophobic effect also contributes to the explanation as to why the CMCs of all 4 AABSs are similar and comparatively higher in the presence of the *trans*-1,3-DACH counterions, as previously discussed. The alteration of parallel and perpendicular binding contributes to a high β in par with the other AABSs/DACH systems. The hypothesized existence of the interstitial space formed upon micellar binding of *trans*-1,3-DACH counterions was further verified by considering the geometry-optimized structures of its diamine precursor obtained by DFT calculations. The resulting optimized structures for all the DACH counterion diamine precursors are presented in Figure 7 and Figure 8 with a +1 and +2 charge.

Based on DFT calculations it was found that most of the DACH counterion diamine precursors have widely protruding amine groups, with the 1,4-DACH counterions having the widest protrusion. As previously stated, is likely that the diamine counterions bind parallel, with the neutral amine hydrogen bonding when the counterion is +1. The only exceptions to this trend are the 1,4-DACH counterions, which support the expected monovalent binding geometry from these diamines as previously explained. The high degree of counterion binding to the micellar superstructure for the 1,4-DACH counterions cannot be explained by the distance between its respective ammonium centers, but rather the higher availability for binding sites due to its perpendicular binding geometry as depicted in Figure 5. For the remaining DACH counterion diamine precursors, the geometrically optimized structures were used to calculate the distance between amine functional groups, as an approximate measure of interstitial space that can form upon counterion condensation to the micelle.

Intermediate distances (4.4–4.9 Å), as in *trans*-1,3-DACH, align well with carboxylate separations at the micelle surface, enabling parallel binding pockets that stabilize aggregation. These geometric constraints explain the unique electrostatic bridging behavior of *trans*-1,3-DACH and the resulting surfactant-independent CMC values.

### 2.4. pH Measurements and Calculations

It should be noted that there is some buffering capacity in the systems from both components: the counterion (DACH), which exists as a mixture of protonation states at the natural pH, and the surfactant, undecanoyl-(headgroup), also contributes to buffering through its ionizable carboxyl group, in the same way amino acids do. To investigate the percentage of counterions in each protonation state, the natural pH of AABS/DACH system, the pKas’ of counterions were utilized in Equations (2)–(4). These equations quantify populations of the doubly protonated (+2), singly protonated (+1), and neutral (0) species. As described in Section 3.8, the resulting distributions are shown in Table 4.

Table 4 shows an estimation of the charge each counterion exhibits in the system as a percentage. For example, in Und-Gly/*cis*-1,2-DACH, 0.06% of the counterions in solution exhibit a +2 charge, while 82.67% exhibit a +1 charge and 17.27% are neutral. Because protonation depends on both pH and pKa, systems where the pH is lower than the pKa, will favor the divalent (+2) form. For example, in the UndAla-/*cis*1,4DACH system, the pH is 9.24 while the pKa is 9.40, resulting in a higher proportion (58.45 %) of divalent counterions. The majority of systems have counterions dominated by a +1 charge (41.51–82.99%). 1,2-, 1,3- DACH systems show that buffering from the DACH^2+^/DACH^1+^ couple is minimal, whereas the DACH^+^/DACH^0^ pair provides useful buffering, the opposite of this is true for 1,4- systems. This effect is likely due to the reduced electrostatic repulsion between the amines in the 1,4-systems, where initial protonation does not significantly hinder subsequent protonation. As a result, the molecule can more readily access multiple protonation states, thereby enhancing buffering in the +2/+1 region.

**Figure 9:**
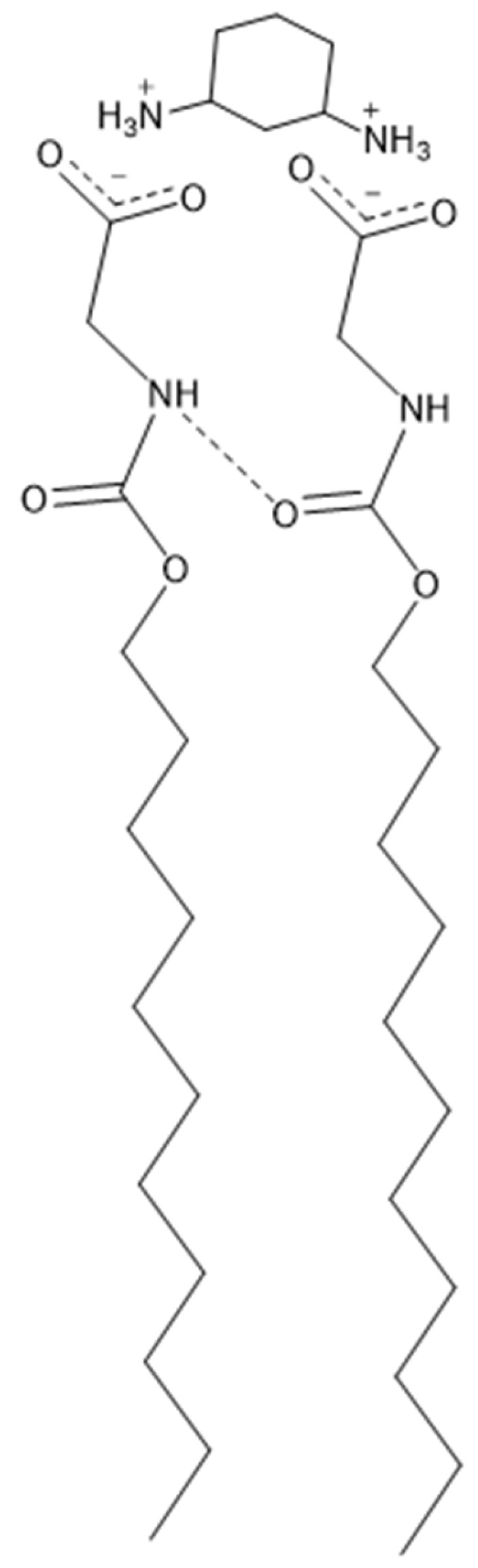
Parallel binding of *trans*-1,3-DACH counterion when exhibiting a +2 charge.

Systems containing *trans*-1,3-DACH have a small percentage of +2 charged counterions, while 1,4-DACH counterions have a much larger percentage (15.27–58.45%). Although most *trans*-1,3-DACH counterions exist in the +1 state at the experimental pH, a small fraction are protonated to the +2 state. Even a minimal amount of divalent counterions can dramatically influence micellar behavior due to their ability to effectively neutralize multiple charges and strong localization at the micellar interface [35,36]. It is likely that the small percentage of divalent counterions in *trans*-1,3-DACH systems have a high binding affinity. The spatial arrangement of amine groups and high binding affinity allows for selective interaction with the most negatively charged regions at the micelle interface first. This geometry favors the formation of parallel binding pockets due to a sufficient distance between amines and enhanced electrostatic complementarity with the carboxylate groups of the surfactant as depicted in Figure 9. This leads to the divalent binding pockets that are sparsely placed, while the remaining positions at the charged interface are then filled by perpendicular binding monovalent *trans*-1,3-DACH counterions or parallel binding monovalent counterions forming binding pockets via hydrogen bonding as explained in Section 2.2. The electrostatic interactions, however, result in stronger binding than the parallel hydrogen bonding interactions, which differentiates the behavior of *trans*-1,3-DACH from that of the other DACH counterions. This stronger stabilization diminishes the influence of the surfactant’s headgroup regardless of whether it is Gly, Ala, Val, or Leu. Because the electrostatic bridging interactions overwhelm the weaker headgroup-dependent forces, the micellization process is governed primarily by the *trans*-1,3-DACH geometry. This causes the energetic cost of micelle formation to become nearly identical across different surfactant headgroups, which explains the consistently similar CMC values observed for *trans*-1,3-DACH systems. The electrostatic bridging dominates the aggregation-driving forces. As a result, CMC values for *trans*-1,3-DACH systems remain consistently similar across different surfactants.

While the protonation state is significant for the *trans*-1,3-DACH counterions, it is less relevant for 1,4-DACH systems. Regardless of the protonation state of the 1,4-DACH counterions, it is likely that the divalent counterions do not contribute to parallel binding pockets due to their protruded geometry as further explained in Section 2.2.

## 3. Materials and Methods

### 3.1. Materials

Starting materials, reagents, and solvents were purchased from commercial suppliers and used without further purification. Deuterated solvents (Cambridge Isotope Laboratories, Tewksbury, MA, USA) for NMR spectroscopic analyses were used as received. Glycine, *L*-alanine, *L*-valine, and *L*-leucine (>97% purity) were purchased from Arcotein, Hoover, AL, USA. Undecanoic acid (>99%), N,N′-diisopropylcarbodiimide (>99%), N-hydroxysuccinimide (NHS; 98%), tetrahydrofuran (THF; ≥99.9%), sodium bicarbonate (≥99.7%), and hydrochloric acid (37%) were all obtained from Sigma-Aldrich, St. Louis, MO, USA. Deionized water with 18.0 mΩ conductivity (Millipore, Bedford, MA, USA) was used for synthesis and solution preparation. The DACH counterion were purchased from the respective manufacturers: *cis*-1,2-DACH (AEchem Scientific Corporation, Downers Grove, IL, USA; >98% purity), (±)-*trans*-1,2-ADCH (Alfa Aesar, Haverhill, MA, USA; 99% purity), *cis*-1,3- DACH (TCI Chemicals, Tokyo, Japan, >98% purity), *trans*-1,3-DACH (TCI > 97.0% purity), *cis*-1,4- DACH (TLC Chemicals, Tokyo, Japan, >98.0%), *trans*- DACH (Aldrich Chemistry, ≥98.0% purity). The filters used are 0.020 µm Whatman PTFE syringe filters.

### 3.2. Analytical Techniques

^1^H-NMR spectra were recorded on a Bruker Avance-II 300 MHz Ultrashield spectrometer (Bruker Corporation, Billerica, MA, USA) at 298 K. Chemical shifts are reported in ppm relative to the residual signal of the solvent. Conductivity measurements were carried out using a Vernier^®^ conductivity probe (Vernier Scientific, Beaverton, OR, USA) and a jacketed beaker connected to a Scientific Caron™ 2050 recirculating bath (Caron Products & Services, Marietta, OH, USA), which maintained the temperature within ±0.05 °C of the set point. Dynamic light scattering (DLS) measurements were performed on a Malvern^®^ Nano Series Zetasizer Nano-ZS (Malvern Panalytical Ltd., Malvern, UK) at a scattering angle (θ) of 173°. pH measurements were taken using a VWR^®^ pHenomenal^®^ pH 1100 L meter (VWR International, Radnor, PA, USA) equipped with a sympHony™ edition 1 pH probe (VWR International, Radnor, PA, USA).

### 3.3. Synthesis of AABSs and Their Respective DACH Counterions

The AABSs were synthesized following previously published methods, whereby undecanoic acid was first coupled with *N*-hydroxysuccinimide (NHS) to form an undecanoyl-NHS ester [21,37]. The undecanoyl-NHS ester was then subjected to amide formation upon coupling with each respective amino acid (Section S1 of the Supplementary Materials). The AABSs were then directly obtained by filtration after having been precipitated from solution with the addition of HCl. The precipitate was washed several times with deionized water to ensure complete removal of HCl residues, then freeze dried. The purity of the AABSs compounds was then verified using ^1^H NMR spectroscopy (Section S2 of the Supplementary Materials).

### 3.4. Variable Concentration Conductivity Measurements

To CMC for each AABS/DACH system was obtained using variable concentration conductivity measurements. In a typical experiment, the conductivity of an initial 10 mL aqueous solution containing equimolar amounts of both AABS the corresponding diaminocyclohexane (40 mM) was measured. These counterions were extracted under nitrogen gas to prevent oxidation. A 1:1 molar ratio of surfactant to counterion was used, as pH-based speciation indicated that the counterion exists mainly in the +1 state, with only a minor fraction of +2 and some neutral species. This distribution results in an effective positive charge that is close to balancing the –1 charge of the surfactant. This solution was then diluted at intervals by removing 1 mL of sample from the solution and adding 1 mL of water via calibrated pipette to return the solution to its original volume. The solution conductivity was measured at each step along this dilution scheme, which was performed until a clear inflection in the linear relationship between conductivity and AABS/DACH concentration could be observed (Section S3 of the Supplementary Materials). This inflection point corresponded to the CMC and was identified from the intersection of two linear trendline equations. The Und-Gly/*trans*-1,4-DACH system could not be measured at room temperature (25 °C) due to poor solubility, even at concentrations significantly below 40 mM, so measurements were instead conducted at 60 °C.

### 3.5. Photon Correlation Spectroscopy/DLS Size Measurements

Micellar size for each AABS/DACH pair was determined using DLS. For each combination of AABS and DACH counterion, a 3 mL aqueous solution was prepared in 0.020 µm filtered deionized water at a concentration that was 5× the CMC at 25 °C. This filtered sample solution was then analyzed by DLS. The highest-intensity peak that was present in both the “by-volume” and “by-intensity” DLS spectra was taken as the average apparent micellar hydrodynamic diameter (Section S4 of the Supplementary Materials). Each sample was prepared and measured in triplicate, with each run consisting of the average of 12 different DLS scans. To explore the temperature-dependent solubility of the Und-Gly/*trans*-1,4-DACH system as mentioned in Section 2.2, hydrodynamic diameters were measured via DLS at 10 different temperatures from 10 to 75 °C. Measurements were taken once the internal Peltier-controlled system in the instrument indicated equilibration to the selected temperature. Based on previous studies, when comparing DLS measurements and DOSY NMR, no significant differences in hydrodynamic size were observed for our system; therefore, no additional measurements were included [38].

### 3.6. Degree of Counterion Binding Calculations

The degree of counterion binding (β) was calculated from the raw variable concentration conductivity data described in Section 2.3. By measuring the change in the slope of the solution conductivity as a function of AABS/DACH concentration, the degree of counterion binding was calculated using Equation (1) and according to previous studies [39]:

(1) $$\beta \;=\frac{m_{B}\;-\;m_{A}}{m_{B}}$$

where *m_B_* is the slope below the CMC and *m_A_* is the slope above the CMC. β represents the fraction of charge-stabilized surfactants due to counterion condensation at the micellar-water interface, assuming no other simultaneous processes influence solution conductivity. Under this regime, β varies from 0 to 1, which, respectively, represent 0% and 100% counterion binding. This parameter, despite its underlying assumptions, is commonly used to compare counterion binding between different surfactant systems [21,22,39,40,41,42,43].

### 3.7. Density Functional Theory Calculations

To provide qualitative insights into the observed experimental results, DFT calculations were performed to identify energy-minimized geometries of the DACH diamine precursors in order to elucidate the binding modes of the DACH counterions using ORCA 6.0 software [44,45]. Hatree-Fock was used for preoptimization, prior to the final run. Then B3LYP-D4 functional was used to correlate DACH electron densities with their overall energies. The D4 component of the B3LYP-D4 functional represents a dispersion correction to the common B3LYP functional that was used to more accurately simulate weak van der Waals forces. This modified functional was used in conjunction with the DEF2-TZVP Opt TightSCF TightOpt basis set [46]. Opt TightSCF and TightOpt were used to ensure tight convergence and optimization. The conductor-like polarizable continuum model (CPCM) was used to implicitly simulate an aqueous environment. This optimization was performed for each DACH counterion diamine precursor and visualized thereafter using the software, Avogadro 2 [47] (Sections S5 and S6 of the Supplementary Materials). It is important to note that DFT calculations provide approximations for the systems being studied rather than direct values.

### 3.8. pH Measurements and Calculations

To investigate pH-dependent protonation of DACH counterions in the system, Equations (2)–(4) were utilized [48] (Section S7 of the Supplementary Materials). The pH measurements were taken at the CMC of each system with triplicate measurements being performed. It should be noted that the pH of the solutions was measured both before and after the CMC measurements, and no significant changes were observed. This stability reflects that the mixtures function as intrinsic buffer systems, since multiple protonation states of the counterions (and of the AABS headgroups) coexist under the experimental conditions. For the Und-Gly and *trans*-1,4-DACH surfactant solution, pH was not measured because the surfactant was not soluble at room temperature. The results from these calculations are presented in Table 4.

(2) $$\alpha_{NH_{3}^{+}-R-NH_{3}^{+}}\;\sim \;\frac{{\left[H^{+}\right]}^{2}}{{\left[H^{+}\right]}^{2}+{\;K}_{a1}[H^{+}]+K_{a1}K_{a2}}$$

(3) $$\alpha_{NH_{3}^{+}-R-NH_{2}}\;\sim \;\frac{K_{a1}[H^{+}]}{{\left[H^{+}\right]}^{2}+{\;K}_{a1}[H^{+}]+K_{a1}K_{a2}}$$

(4) $$\alpha_{NH_{2}-R-NH_{2}}\;\sim \;\frac{K_{a1}K_{a2}}{{\left[H^{+}\right]}^{2}+{\;K}_{a1}[H^{+}]+K_{a1}K_{a2}}$$

Each α represents the fraction of DACH counterions that exist in a given protonation state, expressed as a fraction of the total counterion concentration. The results from these equations can be interpreted qualitatively, as it should be considered that when two pKa values are similar, it is difficult to treat them as distinct protonation states due to overlapping protonation equilibria. Moreover, utilizing DACH pka values for calculations do not account for system specific behavior with the AABSs or micellization.

## 4. Conclusions

In conclusion, this study provides a comprehensive investigation into how stereochemical and constitutional isomerism of DACH counterions modulates the aggregation behavior of AABSs in their natural pH. Through CMC measurements, DLS, DFT calculations, and pH measurements we show evidence suggesting that both the side chain structure of AABSs and the spatial configuration of DACH counterions play pivotal roles in influencing micellar properties such as size, solubility, and degree of counterion binding.

While general trends in micellization were consistent with increasing surfactant hydrophobicity, the *trans*-1,3-DACH counterion uniquely disrupted this pattern, displaying CMCs and β values largely independent of AABS side chain identity. This deviation arises from its structural conformation and pH dependent protonation which showed a small fraction of diavalent counterions. For these counterions, the amine groups are an intermediate distance of ~4.4 Å apart, enabling the small fraction of divalently charged counterions to form strong electrostatic bridging pockets at the micelle interface. As a result, the counterion rather than the surfactant headgroup dictates the CMC. Conversely, the *trans*-1,4-DACH counterion induced abnormal solubility and gel formation in Und-Gly systems, attributed to hindered bidentate binding and enhanced inter-surfactant hydrogen bonding.

These findings suggest that the geometry of the counterion and the structure of the surfactant headgroup are closely linked, highlighting the importance of stereochemical design in controlling micellar systems. Because diamine counterions contain exchangeable hydrogen atoms, their protonation state is sensitive to pH. Consequently, changes in the pH of the surfactant solution can strongly influence the self-assembly behavior of AABSs. This study expands our understanding of surfactant–counterion interactions by introducing a new class of cyclic counterions and suggesting how their structural isomerism may affect micellar organization. In future work, we plan to systematically investigate how pH and ionic strength impact the self-assembly and aggregation properties of AABSs in the presence of diamine counterions.

## Figures and Tables

**Table 1:** CMC of each AABS in the presence of the DACH counterions. ^α^ Conformer (*ae*) refers to axial-equatorial diammonium conformer, and (*ee*) refers to the equatorial-equatorial diammonium conformer, ^β^ Measurements were taken at 60 °C.

Critical Micelle Concentration (CMC) (mM)				
Counterion (Conformer) ^α^	Surfactant			
	Und-Gly	Und-Ala	Und-Val	Und-Leu
*cis*-1,2-DACH (*ae*)	15.9 ± 0.6	14.0 ± 0.2	11.1 ± 0.6	8.0 ± 0.5
*trans*-1,2-DACH (*ee*)	16.6 ± 0.5	15.6 ± 0.4	10.2 ± 0.7	7.4 ± 0.4
*cis*-1,3-DACH (*ee*)	14.3 ± 0.1	9.7 ± 0.8	9.0 ± 0.1	5.9 ± 0.6
*trans*-1,3-DACH (*ae*)	16.0 ± 0.6	13.3 ± 0.8	14.2 ± 0.7	13.2 ± 0.5
*cis*-1,4-DACH (*ae*)	13.3 ± 0.3	13.9 ± 0.2	8.8 ± 0.6	5.8 ± 0.2
*trans*-1,4-DACH (*ee*)	15.1 ± 0.7 ^β^	12.9 ± 0.1	9.5 ± 0.5	4.3 ± 0.2

**Table 2:** The hydrodynamic diameters of AABS micelles in the presence of DACH counterions. ^α^ Measurements were taken at 60 °C.

Size (nm)				
Counterion	Surfactant			
	Und-Gly	Und-Ala	Und-Val	Und-Leu
*cis*-1,2-DACH	2.3 ± 0.2	2.0 ± 0.2	2.0 ± 0.4	2.7 ± 0.6
*trans*-1,2-DACH	1.7 ± 0.2	1.7 ± 0.3	2.0 ± 0.2	2.3 ± 0.2
*cis*-1,3-DACH	2.0 ± 0.6	2.0 ± 0.2	2.7 ± 0.2	2.7 ± 0.2
*trans*-1,3-DACH	1.7 ± 0.2	1.7 ± 0.2	2.3 ± 0.3	2.3 ± 0.4
*cis*-1,4-DACH	2.0 ± 0.2	1.7 ± 0.2	2.7 ± 0.3	2.7 ± 0.3
*trans*-1,4-DACH	4.3 ± 0.3 ^α^	2.7 ± 0.2	2.7 ± 0.2	3.1 ± 0.2

**Table 3:** The degree of counterion binding (β) of each AABS in the presence of DACH counterion. ^α^ Measurements were taken at 60 °C.

Degree of Counterion Binding (β)				
Counterion	Surfactant			
	Und-Gly	Und-Ala	Und-Val	Und-Leu
*cis*-1,2-DACH	0.53 ± 0.02	0.58 ± 0.02	0.42 ± 0.01	0.47 ± 0.01
*cis*-1,3-DACH	0.66 ± 0.02	0.65 ±0.03	0.44 ± 0.01	0.59 ± 0.02
*cis*-1,4-DACH	0.69 ± 0.01	0.59 ± 0.02	0.49 ± 0.02	0.51 ± 0.05
*trans*-1,2-DACH	0.50 ± 0.01	0.52 ± 0.01	0.43 ± 0.01	0.47 ± 0.05
*trans*-1,3-DACH	0.54 ± 0.02	0.56 ± 0.02	0.56 ± 0.05	0.66 ± 0.04
*trans*-1,4-DACH	^α^ 0.75 ± 0.05	0.61 ± 0.01	0.48 ± 0.02	0.64 ± 0.02

**Table 4:** Calculated protonation state distributions (2^+^, 1^+^, 0) for AABS systems paired with various DACH counterions at natural pH. Values represent the mole fraction of each protonation state, calculated using Equations (2)–(4). * no data due to limited solubility at room temperature.

Surfactant	DACH Isomer	pH	pKa_1_	pKa_2_	+2%	+1%	0%
Und-Gly	*cis*-1,2-DACH	9.25 ± 0.05	6.13 [23]	9.93 [23]	0.06	82.67	17.27
Und-Ala		9.24 ± 0.09			0.06	82.99	16.94
Und-Val		9.44 ± 0.02			0.04	75.52	24.44
Und-Leu		9.27 ± 0.02			0.06	82.00	17.91
Und-Gly	*trans*-1,2-DACH	9.66 ± 0.01	6.47 [23]	9.94 [23]	0.04	65.55	34.4
Und-Ala		9.62 ± 0.01			0.05	67.6	32.35
Und-Val		9.71 ± 0.00			0.04	62.92	37.05
Und-Leu		9.61 ± 0.02			0.05	68.1	31.85
Und-Gly	*cis*-1,3-DACH	10.33 ± 0.04	8.29 [34]	10.3 [34]	0.44	48.06	51.5
Und-Leu		10.21 ± 0.07			0.66	54.8	44.54
Und-Val		10.31 ± 0.03			0.47	49.19	50.34
Und-Ala		10.33 ± 0.13			0.44	48.06	51.5
Und-Gly	*trans*-1,3-DACH	9.72 ± 0.02	8.54 [34]	10.36 [34]	5.10	77.21	17.69
Und-Ala		9.55 ± 0.03			7.80	79.83	12.36
Und-Val		9.90 ± 0.04			3.14	71.92	24.94
Und-Leu		10.03 ± 0.01			2.16	66.66	31.18
Und-Gly	*cis*-1,4-DACH	9.26 ± 0.07	9.4 [23]	10.8 [23]	57.30	41.51	1.2
Und-Ala		9.24 ± 0.10			58.45	40.44	1.11
Und-Val		9.44 ± 0.05			46.63	51.13	2.23
Und-Leu		9.28 ± 0.03			56.13	42.58	1.29
Und-Gly *	*trans*-1,4-DACH		9.4 [23]	10.8 [23]			
Und-Ala		9.93 ± 0.07			20.64	69.93	9.43
Und-Val		10.07 ± 0.07			15.27	71.43	13.3
Und-Leu		9.86 ± 0.07			23.72	68.42	7.86

## Data Availability

The original contributions presented in this study are included in the article/Supplementary Materials. Further inquiries can be directed to the corresponding authors.

## Supplementary Materials

The following supporting information can be downloaded at: https://www.mdpi.com/article/10.3390/molecules30204114/s1, Figure S1: Synesthetic scheme for undecanoyl-AABSs. Further information can be obtained on request; Figure S2: Und-Gly ^1^H NMR spectrum in CD_3_OD; Figure S3: Und-Ala ^1^H NMR spectrum in CD_3_OD; Figure S4: Und-Val ^1^H NMR spectrum in CD_3_OD; Figure S5: Und-Leu ^1^H NMR spectrum in CD_3_O; Figure S6: Conductivity vs. concentration data for Und-Gly, Und-Ala, Und-Val, and Und-Leu in the presence of *cis*-1,2-DACH at room temperature. Each point represents the average of three triplicate measurements. Similar trends were observed in the presence of other counterions. Full replicated data is available on request; Figure S7: Size distribution curve by intensity of 12 runs of Und-Leu with *cis*-1,3-DACH triplicate three taken at room temperature and 5x the CMC; Figure S8: Size distribution curve by volume of 12 runs of Und-Leu with *cis*-1,3-DACH triplicate three taken at room temperature and 5x the CMC; Section S1: Synthetic Scheme; Section S2: NMR Spectrums for Undecanoyl-AABSs; Section S3: CMC: Conductivity vs. Concentration Plots; Section S4: DLS Size Distribution Plots; Section S5: ORCA Geometry Optimization; Section S6: ORCA Geometry Optimized Coordinates; Table S1: Optimized systems: atom counts and calculated electronic energies of DACH counterions; Section S7: Derived pH Protonation Equation: Equations (S1)–(S23).

## Abbreviations

The following abbreviations are used in this manuscript:

AABS	Amino Acid-Based Surfactant
DLS	Dynamic Light Scattering
CMC	Critical Micelle Concentration
DACH	Diamino Cyclo Hexane
Und-Gly	Undecanoyl-Glycine
Und-Ala	Undecanoyl-L-Alanine
Und-Val	Undecanoyl-L-Valine
Und-Leu	Undecanoyl-L-Leucine
DFT	Density Functional Theory
