# Supplementary material for: Stereoisomeric Effects of Diammoniumcyclohexane Counterions on the Self-Assembly of Amino Acid-Based Surfactants
Source: Molecules. 2025 Oct 16;30(20):4114. doi: 10.3390/molecules30204114 (PMC12566052; doi:10.3390/molecules30204114)
Supplement: Supplementary file 1 [file molecules-30-04114-s001.zip › molecules-3863154-supplementary.pdf]

Supplementary Materials for:

Stereoisomeric Effects of Diammoniumcyclohexane Counterions on the Self-Assembly of Amino Acid-Based Surfactants

Saylor E. Blanco [1], Nathan Black [2,*], Margarita A. Alvarez [1], Kevin F. Morris [3], Mark A. Olson [1], Eugene J. Billiot [1] and Fereshteh H. Billiot [1,*]

[1] Department of Physical and Environmental Sciences, Texas A&M University Corpus Christi, 6300 Ocean Dr., Corpus Christi, TX 78412, USA; sblanco3@islander.tamucc.edu (S.E.B.); malvarez20@islander.tamucc.edu (M.A.A.); mark.olson@tamucc.edu (M.A.O.); eugene.billiot@tamucc.edu (E.J.B.)
[2] Department of Chemistry, University of Washington, 4000 15th Avenue NE, Seattle, WA 98195, USA
[3] Department of Chemistry, Carthage College, 2001 Alford Park Drive, Kenosha, WI 53140, USA; kmorris@carthage.edu
* Correspondence: nblack1@uw.edu (N.B.); fereshteh.billiot@tamucc.edu (F.H.B.)

Tables of Contents

Section S1. Synthetic Scheme……………..…………………………………….......Page 2-3

Section S2. NMR Spectrums for Undecanoyl-AABSs……………………………......Page 3-6

Section S3. CMC: Conductivity vs. Concentration Plots…………………………..…..Page 7

Section S4. DLS Size Distribution Plots………………………………..…..................Page 8-9

Section S5. ORCA Geometry Optimization …………………….......................……..Page 9-11

Section S6. ORCA Geometry Optimized Coordinates……………...……..…………...Page 12-19

Section S7. Derived pH Protonation Equation……………...…...…………………..……Page 19-23

Section S1. Synthetic Scheme

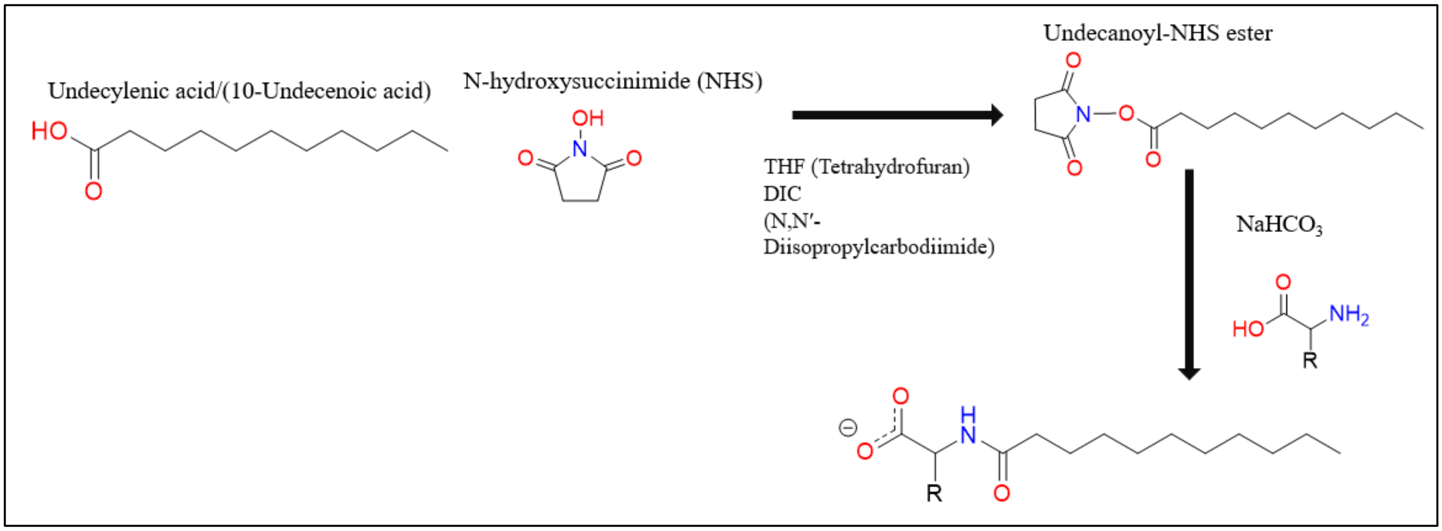

Figure S1. Synesthetic scheme for undecanoyl-AABSs. Further information can be obtained on request.

Undecanoic acid is first activated by conversion into the undecanoyl-NHS ester using N-hydroxysuccinimide (NHS). NHS is dissolved in a minimal amount of THF (tetrahydrofuran) which is coupled with undecanoic acid solubilized in DIC (diisopropylcarbodiimide). There are two methods for preventing exothermic reactions, (1) ice bath while adding, (2) adding small aliquots at a time. After the addition it was allowed to react for 48+ hours. The reaction mixture was first filtered to remove the insoluble DIC urea byproduct, and the solvent THF was removed by rotary evaporation. The crude product was transferred into warm 2-propanol, clarified by heating, and then left in the freezer overnight to induce solid formation. The resulting solid was scraped, cut into small pieces, and repeatedly washed with cold 2-propanol and MilliQ water to remove residual NHS and DIC. The purified product was then collected by vacuum filtration, freeze-dried, weighed to determine yield, and analyzed by ^{1}H NMR spectroscopy.

Once the NHS ester is formed, it reacts with the amino acid derivative (R-NH_2) in 2.0 molar excess to the und-NH easter in THF as a solvent, producing the final undecanoyl-AABS surfactant through amide bond formation. Not noted in this figure is that the AABSs were then directly obtained by filtration after having been precipitated from solution with the addition of HCl. The precipitate was washed several times with deionized water to ensure complete removal of HCl residues, then freeze dried. The purity of the AABSs compounds was then verified using ^{1}H NMR spectroscopy.

Section S2. NMR Spectrums for Undecanoyl-AABSs

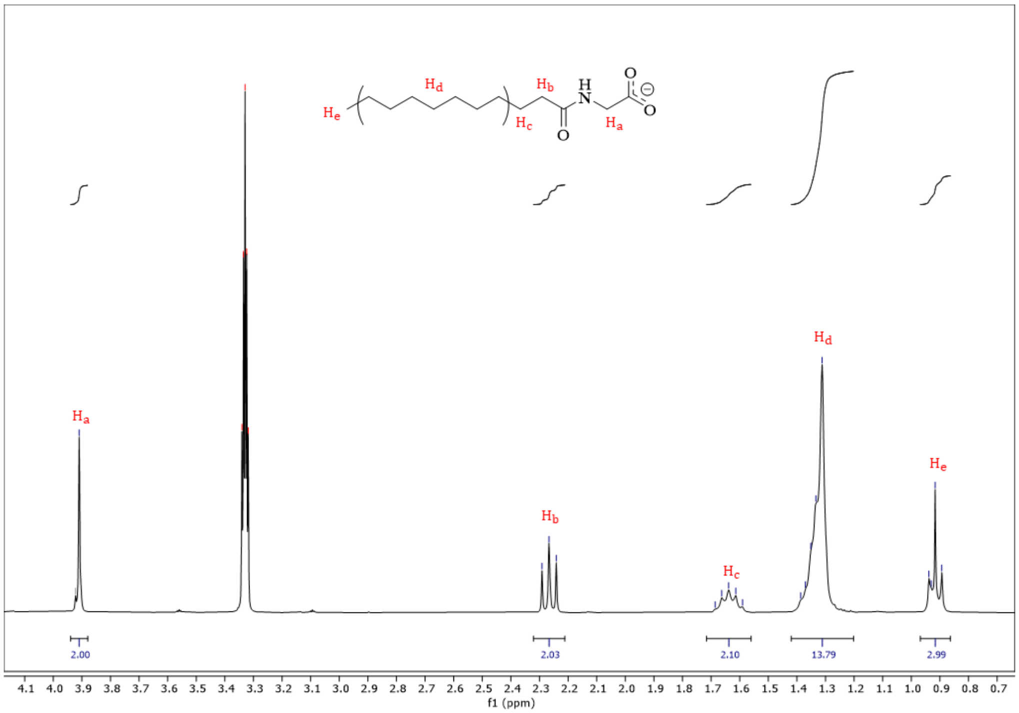

Figure S2. Und-Gly ^{1}H NMR spectrum in CD3OD.

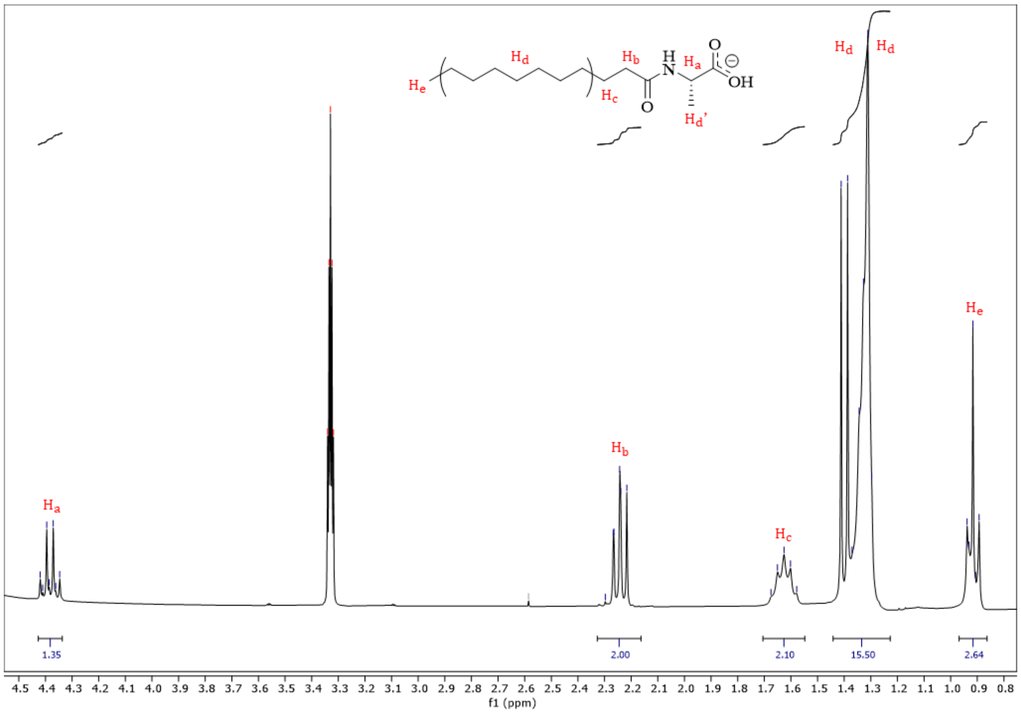

Figure S3. Und-Ala ^{1}H NMR spectrum in CD3OD.

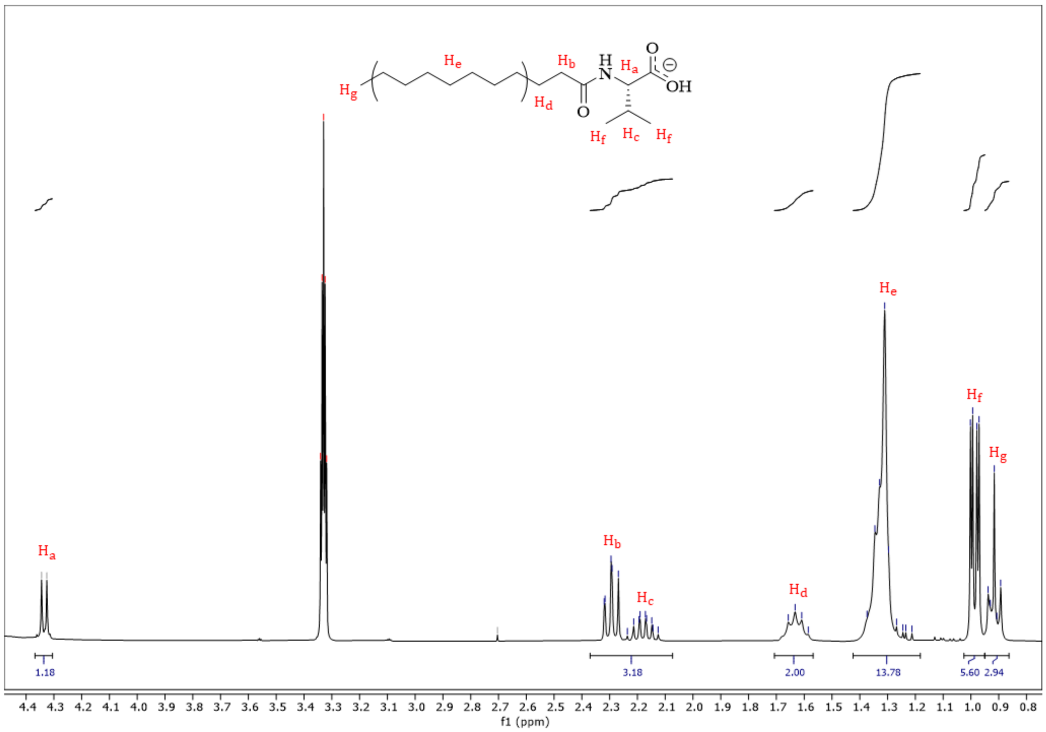

Figure S4. Und-Val ^{1}H NMR spectrum in CD_3OD.

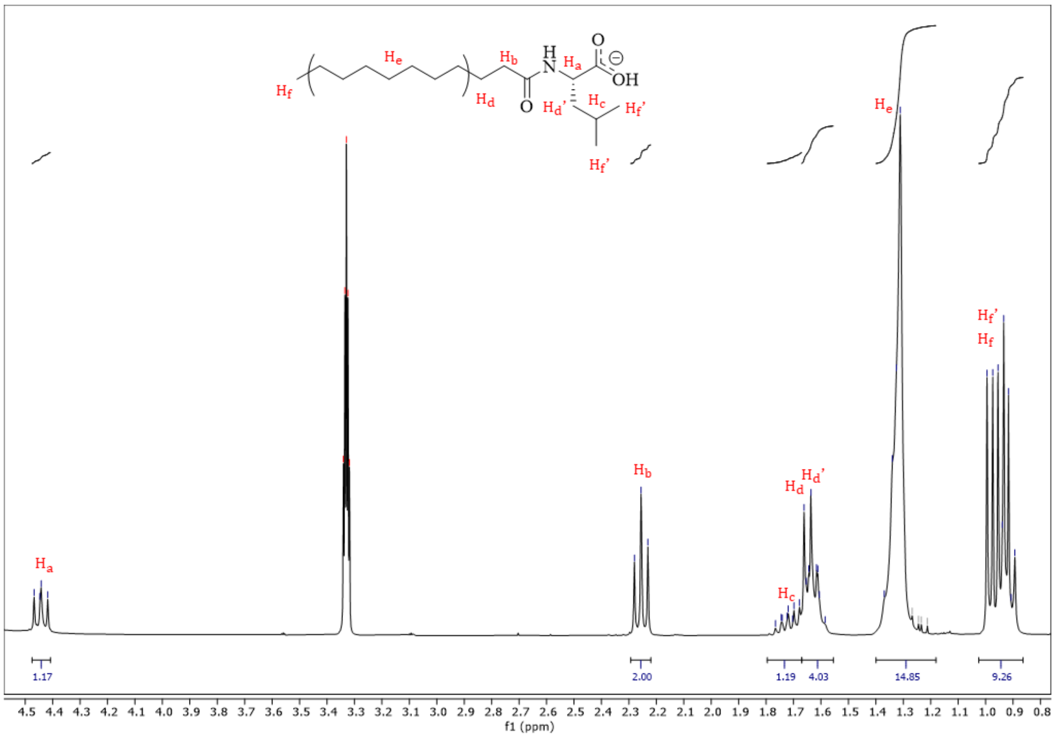

Figure S5. Und-Leu ^{1}H NMR spectrum in CD_3OD.

Section S3. CMC: Conductivity vs. Concentration Plots

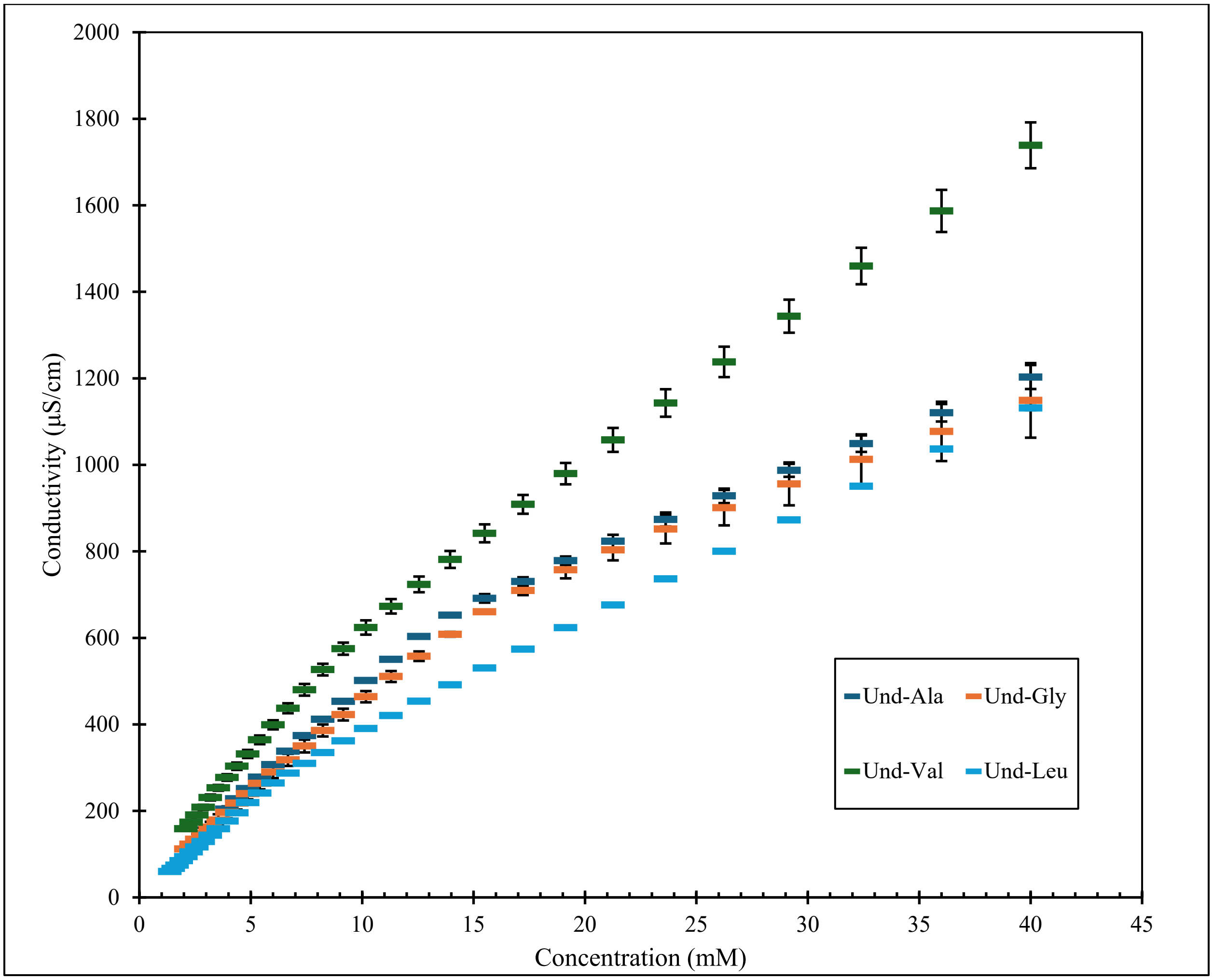

Figure S6. Conductivity vs. concentration data for Und-Gly, Und-Ala, Und-Val, and Und-Leu in the presence of cis-1,2-DACH at room temperature. Each point represents the average of three triplicate measurements. Similar trends were observed in the presence of other counterions (not shown). Full replicated data is available on request.

Section S4. DLS Size Distribution Plots

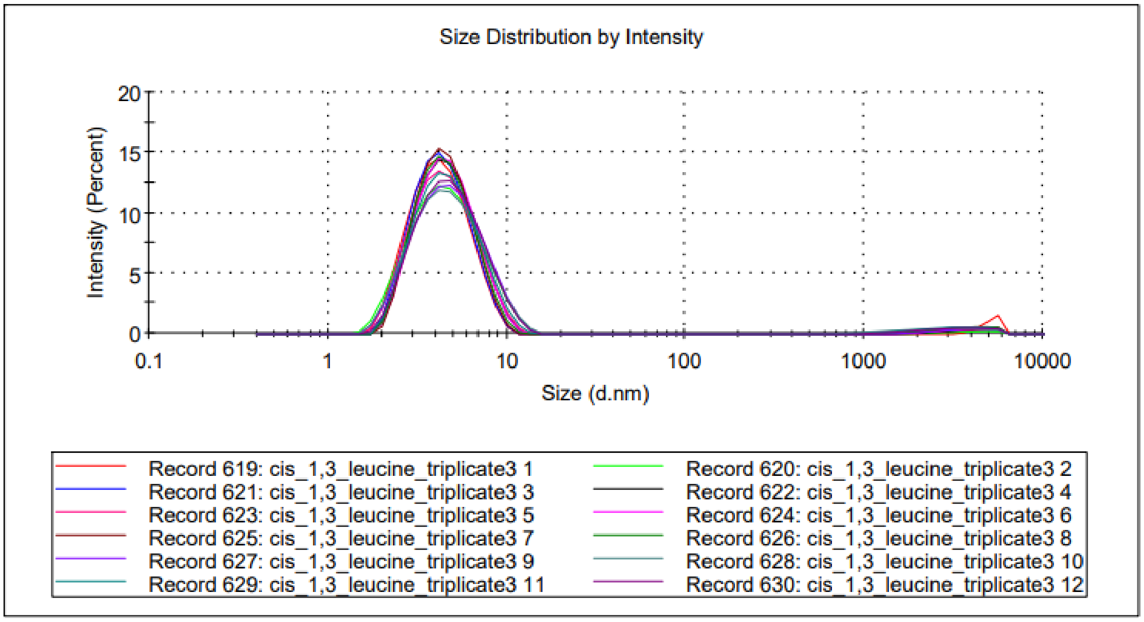

Figure S7. Size distribution curve by intensity of 12 runs of Und-Leu with cis-1,3-DACH triplicate three taken at room temperature and 5x the CMC.

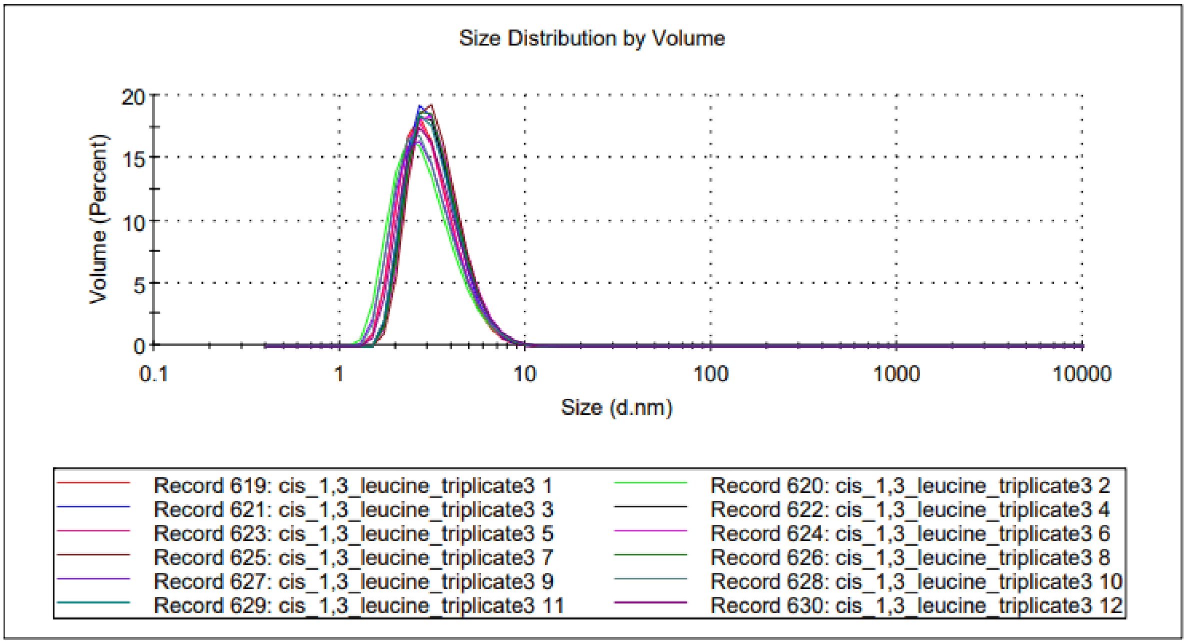

Figure S8. Size distribution curve by volume of 12 runs of Und-Leu with cis-1,3-DACH triplicate three taken at room temperature and 5x the CMC.

Section S5. ORCA Geometry Optimization

Note: The optimized coordinates are saved in an external file: DFT_Optimized_Coordinates.pdf

All optimized structures were confirmed as minimum by frequency analysis (no imaginary frequencies).

System	Atoms	Energy (Eh)
C12 High pH	23	-346.989498036890
C21 Low pH	24	-347.443547260923
C13 High pH	23	-346.989569825350
C13 Low pH	24	-347.453119175087
C14 High pH	23	-346.979793135178
C14 Low pH	24	-347.444043783202
T12 High pH	23	-346.980414272992
T12 Low pH	24	-347.443546317216
T13 Low pH	24	-347.451650145363
T13 High pH	23	-346.987498028491
T14 High pH	23	-346.986961166951
T14 Low pH	24	-347.454659591864

Table S1. Optimized systems: atom counts and calculated electronic energies of DACH counterions.

Preoptimization Input File

```
! HF Opt


%pal

  nprocs 9

end


%maxcore 1000


# xyz 2 1 for low pH

# xyz 1 1 for high pH

[coordinates inserted from Avogadro]
```

Optimization Input File

```
! B3LYP D4 DEF2-TZVP Opt TightSCF TightOpt Freq


%method
  GridX 5
end


%pal
  nprocs 9     # adjust based on your CPU cores
end


%maxcore 1000  # adjust based on available RAM per core


%cpcm
  epsilon 78.4
end
# xyz 2 1 for low pH
# xyz 1 1 for high pH


[coordinates inserted from preoptimization output]
```

Section S6. ORCA Geometry Optimized Coordinates

Optimized Cartesian Coordinates: Cis-1,2-DACH High pH

C	0.51314363962704	3.41793835950148	-0.34961978598799
C	-0.84756274270025	3.38508493997705	0.34830773376556
C	-1.58363363033794	2.07437809762666	0.06271520767793
C	-0.75064846227093	0.84228444592833	0.43631803301292
C	0.60466505789389	0.91851153452154	-0.27953722887956
C	1.36380791971207	2.20257133464297	0.02684722293569
N	1.41280072235890	-0.27092807990336	0.15525147552644
N	-0.52154063390070	0.65155101367503	1.87690256900229
H	0.36441547038428	3.42819947956624	-1.43463625781921
H	1.05300899108417	4.33185559954009	-0.09379537881274
H	-1.45956278606522	4.22811663166061	0.01991300430776
H	-0.70632915654782	3.50856184739922	1.42746440721506
H	-1.82133780441117	2.01998064717447	-1.00484545380569
H	-2.53180230371760	2.03410689909488	0.60399093547928
H	-1.26656935613023	-0.05258536750339	0.08219291286951
H	0.45557705513064	0.82057575812590	-1.35384425287106
H	2.30725327448046	2.20599572938129	-0.52447544337676
H	1.61282419831526	2.23847702669377	1.09221140700318
H	1.28725085758648	-0.35989120989247	1.17270193540702
H	2.40286313564478	-0.16225033767456	-0.06807151914647
H	1.07262138945369	-1.12804673012327	-0.28352602192040
H	-0.30837187675067	1.53621087277441	2.32542557570744
H	-1.36849295883914	0.30031150781307	2.30811892270977

Optimized Cartesian Coordinates: Cis-1,2-DACH Low pH

C	0.49448997448438	3.40555450616237	-0.22051826749581
C	-0.92965656731413	3.37744932616746	0.33580798652761
C	-1.64993551525646	2.07542207309698	-0.02536186950844
C	-0.87168322871122	0.83066904477581	0.40235473933176
C	0.54424963569578	0.90862756609596	-0.17180353641956
C	1.29787549173953	2.17506114839612	0.21080474031870
N	1.36545379694774	-0.31433842613447	0.13853181220434
N	-0.90898310855449	0.71764751370377	1.91159804269626
H	0.45651049845727	3.43842582782979	-1.31371129116315
H	1.01255730923026	4.30724184125498	0.10969069790679
H	-1.50360959045131	4.21806675274975	-0.05784949336615
H	-0.90229895680335	3.50755571081125	1.42263607008203
H	-1.77944204006042	2.01117595238771	-1.10780312912743
H	-2.64699545872114	2.03913459098079	0.41814360533862

H	-1.36523497478473	-0.07083069191938	0.04300269503377
H	0.40989629667121	0.88309357108068	-1.25305400773570
H	2.27438225194125	2.16612393103880	-0.27711728521001
H	1.47863706719051	2.19576309551871	1.29038759007673
H	1.63538154673551	-0.36242305948424	1.12562028253833
H	2.23083153325253	-0.29571367288557	-0.40764592872953
H	0.86066964785089	-1.17250055774134	-0.09888918919127
H	-0.44225955073709	1.49788382289589	2.37963967974099
H	-0.48619417951188	-0.14872535267067	2.25377483425141
H	-1.88565187929064	0.71827548588883	2.21737122189970

Optimized Cartesian Coordinates: Cis-1,3-DACH High pH

C	0.21030004459130	2.34078648753034	0.43209881806828
C	-1.11689110383416	2.55061554055480	-0.29854184699572
C	-1.89834756420712	1.24391424728088	-0.46096630468812
C	-1.03488533040819	0.18869146062444	-1.15031493959203
C	0.27887649584211	-0.00817895156031	-0.40879323185149
C	1.06806806617065	1.28376349987172	-0.26510888387005
N	1.10945914982716	-1.02700681464341	-1.14426284296966
N	-3.16004313029976	1.39104857189001	-1.19855159143635
H	0.76439295000334	3.27957440911143	0.49017183577672
H	0.01510203460766	2.02466576539149	1.46212866608908
H	-1.73469884899279	3.27443461011749	0.23994268618384
H	-0.92689146398021	2.96578915602095	-1.29531508545627
H	-2.15204608790065	0.87272236810985	0.53708501183609
H	-1.57725270808037	-0.75798409075938	-1.20730648883530
H	-0.82217480858292	0.51341015142779	-2.17585204384103
H	0.09275845840962	-0.44371035519986	0.57353803237529
H	1.35487388073866	1.63713964414169	-1.26142749252433
H	1.98632691464254	1.09729387369101	0.29729991748419
H	1.31874357056452	-0.70159135014693	-2.09153809490408
H	0.61323976360360	-1.91792478027366	-1.21806179545423
H	1.99651814422356	-1.19243133817218	-0.66381008288745
H	-2.95115558777699	1.77828504439209	-2.11616580159039
H	-3.72928283916155	2.09092285059974	-0.73063844091695

Optimized Cartesian Coordinates: Cis-1,3-DACH Low pH

C	0.20735975947636	2.34102408069187	0.42688485711289
C	-1.12702938553626	2.55611887908960	-0.28913022919673
C	-1.86771955691414	1.23422808029481	-0.42740599139281
C	-1.03873880792553	0.17696907903601	-1.14470681283438
C	0.28012195580138	-0.01441872791383	-0.40666388638095
C	1.06128502696721	1.28486416947773	-0.27661204464667
N	1.10398927336519	-1.03250431190784	-1.14416845182533

N	-3.14546848995058	1.44619162071774	-1.18982633120959
H	0.75384877498427	3.28358493204841	0.47122630226631
H	0.02152183299838	2.03033031633511	1.45926934852565
H	-1.74517688601633	3.26857914896705	0.26119707175314
H	-0.94984424556211	2.96956256548262	-1.28728474008779
H	-2.17101501902596	0.86765575197634	0.55342339515799
H	-1.58766939558118	-0.76631282542129	-1.19187654891724
H	-0.83594839338917	0.50696077806902	-2.16896595083392
H	0.09827468056364	-0.44449803308198	0.57839647673383
H	1.33779636641546	1.63510881539201	-1.27640948634628
H	1.98342201688975	1.10623356627097	0.28066246271289
H	1.30915448252111	-0.71149091176898	-2.09422666258342
H	0.61069425755913	-1.92608700722923	-1.21020634080391
H	1.99387096237096	-1.19282291138178	-0.66657594599566
H	-2.95140852588326	1.79936879740626	-2.13057578457438
H	-3.74264972471051	2.12621759513443	-0.71396358529385
H	-3.66809095941783	0.57145655231495	-1.27985112133975

Optimized Cartesian Coordinates: Cis-1,4-DACH High pH

C	0.30013499851231	3.70173177574373	0.78373250788467
C	-1.04410847095166	3.76258157332685	0.03479832794385
C	-1.53566067879300	2.38428367269951	-0.43711617047367
C	-1.07129689825932	1.29916812647595	0.53687140625661
C	0.46452640891199	1.18886760985439	0.57278486759284
C	1.12745365483897	2.53666088216866	0.26863422328001
N	2.49895726194170	2.56705587889897	0.88767240240681
N	-2.98805116272500	2.43108341761066	-0.64636240611731
H	0.13483925862893	3.56213499987757	1.85587334338411
H	0.84684671968243	4.63896034426676	0.66143825516812
H	-1.79628730875842	4.19249885646986	0.70138727942489
H	-0.96460782478012	4.42916640811532	-0.82607610777946
H	-1.08747940308514	2.16851673899207	-1.41170120938139
H	-1.50172441666330	0.33704527761937	0.25229612620247
H	-1.45633629822233	1.53845698024534	1.53309858184287
H	0.81450017589635	0.44923888337905	-0.14954513005268
H	0.78256166480054	0.84773570245397	1.56180109497470
H	1.30038329578333	2.64871083748564	-0.80114143986879
H	2.43238275572999	2.50656855661828	1.90710573310463
H	2.98896929560509	3.43218163954056	0.65075272821489
H	3.06418709075977	1.77969757790563	0.56284866120198
H	-3.43731652091075	2.59205844064752	0.25228851783583
H	-3.30582359794234	1.51655581960431	-0.95542159304596

Optimized Cartesian Coordinates: Cis-1,4-DACH Low pH

C	0.30254345071601	3.70793099774025	0.79410772742623
C	-1.03709131802961	3.78774552609119	0.04043247675140
C	-1.51129452832264	2.40329516629073	-0.42064758129095
C	-1.07717321450649	1.30517765275634	0.53678918127410
C	0.45770738100465	1.19387500092252	0.55092490859539
C	1.12725471572049	2.54461689024934	0.26756598126287
N	2.49460923156514	2.56168927383456	0.89123613730704
N	-3.00894042305862	2.40617499594774	-0.55035161959429
H	0.12905448082621	3.55990231347280	1.86298446190248
H	0.84819894825102	4.64549555221633	0.67857266406444
H	-1.78640217195795	4.22651983163506	0.70300990000567
H	-0.94693818553430	4.44200543765818	-0.82729834037151
H	-1.14244326131321	2.18719177057935	-1.42274402473231
H	-1.51474804321188	0.34967630097268	0.24388376781280
H	-1.45000484817096	1.54330590100663	1.53620742392560
H	0.79375540418619	0.46612472349238	-0.18846000776561
H	0.77403630162435	0.83154512944443	1.53166257952172
H	1.30287198812837	2.66888630433689	-0.80053146877894
H	2.42612047604171	2.49237416823501	1.91022184742243
H	2.98960782941439	3.42654609095317	0.66281321010063
H	3.05717547659762	1.77477660765407	0.56003463406660
H	-3.44989591660087	2.56591053265479	0.35922375016264
H	-3.34567619170914	1.51222960048990	-0.91500762427468
H	-3.31973758166041	3.14659423136558	-1.18375998479375

Optimized Cartesian Coordinates: Trans-1,2-DACH High pH

N	-1.13822703842902	2.84960795927346	1.23820742339062
C	-1.35329584892166	2.49370171333016	-0.20318981333802
C	-1.04220462513582	1.01353687137676	-0.44952578853593
C	-0.57414266995249	0.76373166784840	-1.89431696491765
C	-1.23334666295673	1.74544960161902	-2.86136309506535
C	-0.82477893420084	3.19702877062924	-2.54197539219247
C	-0.49122910704967	3.42438290878416	-1.05559971533964
N	-0.65759917637371	4.80895148657207	-0.59758093175535
H	-1.10454325119753	3.87713941697998	1.29143223934406
H	-0.24384484947739	2.48463604700300	1.57326566913478
H	-1.87878980116650	2.48437582822430	1.83717344969709
H	-2.40478498400491	2.71083934608443	-0.39544987917981
H	-0.26416932014575	0.68940013727597	0.24702339877903
H	-1.93339379611133	0.42319782738963	-0.22998222623497
H	0.51198240233253	0.87459745901383	-1.95492467321458
H	-0.79950153040286	-0.26642096891659	-2.17501510333772
H	-0.96945707143097	1.50313300517858	-3.89238322509621
H	-2.31980452913554	1.64128777895469	-2.78523828427656
H	-1.63645273282631	3.87616416901914	-2.81730895637059

H	0.04551152627305	3.48744953430006	-3.13437677298994
H	0.55289692329754	3.15732739961299	-0.87327010046073
H	-1.48964343433423	5.20941035712556	-1.02189707395987
H	0.12735851135020	5.36974168332111	-0.90847418408015

Optimized Cartesian Coordinates: Trans-1,2-DACH Low pH

C	0.49434178720321	3.40594802757626	-0.21933866178720
C	-0.93048090582779	3.37695027190449	0.33525195797665
C	-1.64969616747861	2.07467982355339	-0.02704293774567
C	-0.87133229045900	0.83022361453674	0.40126593981291
C	0.54510819246748	0.90906305598386	-0.17137401246554
C	1.29775545350809	2.17566561863051	0.21251687329473
N	1.36657252203325	-0.31363198911331	0.13951436353527
N	-0.91015993268096	0.71694415826076	1.91048754493607
H	0.45770318470023	3.43926764391523	-1.31256056971332
H	1.01150283283711	4.30778415093531	0.11189179167499
H	-1.50441147791652	4.21735003366777	-0.05893106931288
H	-0.90449616918904	3.50698938501751	1.42212199224473
H	-1.77780806178400	2.01066588987712	-1.10966399895077
H	-2.64727744030247	2.03788947613444	0.41522539274403
H	-1.36409857600010	-0.07146318688342	0.04133397303054
H	0.41188840176810	0.88377414956399	-1.25276223203441
H	2.27488263560186	2.16724925135979	-0.27417540563056
H	1.47725021394399	2.19608753884047	1.29231517703870
H	1.63538087330549	-0.36182870189099	1.12690402860763
H	2.23259041670771	-0.29417340621019	-0.40562809267302
H	0.86258493157482	-1.17196362740775	-0.09894608816130
H	-0.44505714448870	1.49781511551883	2.37909547932407
H	-0.48654337723972	-0.14885943289504	2.25306964436458
H	-1.88720990228444	0.71621313912417	2.21503890988978

Optimized Cartesian Coordinates: Trans-1,3-DACH High pH

C	0.71478510774106	1.33537588441437	-0.10713447036427
C	-0.78787231555405	1.35594573607284	0.17953323362712
C	-1.51455212855530	0.12089056379895	-0.34201814404847
C	-0.82420825054334	-1.18149377179732	0.05234655435080
C	0.67512670411405	-1.15769598941735	-0.23223588831127
C	1.36367504598864	0.04485006341569	0.39657387651288
N	1.28351353351432	-2.42929265960846	0.29015196561689
N	-1.66393934485385	0.19885250810775	-1.84688338356150
H	1.18531475015792	2.19400732483426	0.37326677663293
H	0.89979039571515	1.44076843781117	-1.18078338305841
H	-0.95187548363626	1.38012511062662	1.25933666251466
H	-1.25177842508377	2.25471270395738	-0.23298355164611

H	-2.53701398096134	0.10499417190501	0.02819930158014
H	-1.29774765548762	-2.02848223825941	-0.44958968610228
H	-0.97876932182841	-1.30763129840408	1.12696991347033
H	0.86601355069293	-1.17542544430045	-1.30583591272935
H	2.42807520729628	0.03027150290602	0.15296360107527
H	1.26939778965462	-0.01902899005854	1.48579463214235
H	1.15727730932434	-2.50142052512885	1.30363496656050
H	2.28710744083340	-2.45763769175914	0.09550828291754
H	0.84998892395683	-3.24879889487256	-0.14190458000729
H	-0.76331205435521	0.22338148453873	-2.33056437254336
H	-2.17706430003300	1.04355606621925	-2.10747446339445
H	-2.18270249809741	-0.60900405500188	-2.19764193123466

Optimized Cartesian Coordinates: Trans-1,3-DACH Low pH

C	0.84420429808930	1.33915762181275	-0.03663424813109
C	-0.67138137324816	1.40552387220543	0.16109962777798
C	-1.41389297309513	0.22251040640731	-0.46590075201280
C	-0.80817525252540	-1.10576967249317	-0.00361912013248
C	0.69685158054473	-1.14709083920144	-0.22931856982966
C	1.41285441004691	0.00673985049979	0.45519626591075
N	1.23478840788329	-2.45307606826796	0.29534105004415
N	-1.36471219741671	0.30775186794664	-1.93565717213780
H	1.32423738194305	2.15841794619901	0.50243273008333
H	1.09196324711217	1.46913338146597	-1.09276295626151
H	-0.89385133853192	1.41600976039441	1.23249065322376
H	-1.06672771357780	2.33750647430934	-0.25280865299016
H	-2.44837291836825	0.24624307629642	-0.10263867247308
H	-1.28945295358494	-1.93665101843826	-0.52738049285127
H	-1.00922377428462	-1.23000120973737	1.06496422844231
H	0.92394384724071	-1.15220748353320	-1.29378268632064
H	2.48635882388095	-0.05145663565189	0.25892118943157
H	1.26975068067558	-0.08064761026405	1.53818218002913
H	1.06785029325185	-2.53306169552372	1.30176056284162
H	2.24246189596835	-2.52332805678115	0.13786914264959
H	0.78149351201692	-3.24613064077104	-0.16391795997873
H	-1.80910492787414	1.17262270014574	-2.22815188246935
H	-1.92159516344673	-0.44528336011957	-2.32830659784559

Optimized Cartesian Coordinates: Trans-1,4-DACH High pH

N	1.52445066416060	-0.35226432626123	0.70541098927840
C	0.30912858888730	-0.57031107456375	-0.09938441113154
C	0.14605858168299	-2.00987658045468	-0.61246262394287
C	1.18132166733818	-2.37041890335542	-1.68203035125032

C	1.22736992145080	-1.37342020226129	-2.83621746364335
N	0.01939565483699	-1.56479946673691	-3.73180466665488
C	1.29656281073493	0.07491424944349	-2.35975873172897
C	0.26347127006583	0.40651978758291	-1.27708414898136
H	2.34936580195381	-0.54457101551917	0.14517244520916
H	1.54292789458259	-1.03617839719388	1.45656482951748
H	-0.53768384176550	-0.34998458337501	0.55397492940127
H	0.23254178177573	-2.70912357038353	0.22276500590139
H	-0.86689365429216	-2.12927712805296	-1.01210772189478
H	2.18132928973703	-2.38077914402548	-1.24214721653892
H	1.00434287792356	-3.37702188535778	-2.06873339492598
H	2.07434533427976	-1.59269868141534	-3.48368095582401
H	-0.85798964422054	-1.37832723698893	-3.24140233136851
H	-0.01780695867286	-2.52506817836281	-4.07946533115219
H	0.06629953716394	-0.93500535381063	-4.53525429636355
H	1.18668086175523	0.74979370900113	-3.21211026265874
H	2.30748292497114	0.22937576408991	-1.97537839546631
H	-0.74333658364376	0.38502207584513	-1.70492430297712
H	0.42807521929444	1.42553014215626	-0.92168159280424

Optimized Cartesian Coordinates: Trans-1,4-DACH Low pH

N	0.88341564152240	-0.22046569763109	1.28235957412817
C	0.42844604321520	-0.58327100189170	-0.10445356591652
C	0.88278129365908	-1.99950980117895	-0.43173676619620
C	0.47147381200665	-2.37378794545652	-1.85534455974087
C	1.01223787207536	-1.35235941828221	-2.84666546791011
N	0.55746054648037	-1.71522140125358	-4.23351409597797
C	0.55784733898886	0.06385963740721	-2.51942731073690
C	0.96911777953115	0.43816398948185	-1.09581340607694
H	0.57249134814446	0.72074223317178	1.53337566038308
H	1.90483931164917	-0.24322300730143	1.34568679761909
H	0.50920772285705	-0.87538815431153	1.97253693121051
H	-0.66042540799345	-0.53690989339909	-0.07521355197363
H	1.97160559661090	-2.05670842418496	-0.33398911404345
H	0.44919913681940	-2.70668843172129	0.27751822377895
H	0.84693758982082	-3.36921454394370	-2.09911570837434
H	-0.62016192040161	-2.40229681999231	-1.93096539964844
H	2.10111069074713	-1.39874730779110	-2.87576052426618
H	-0.46397169374507	-1.69299984463539	-4.29687050155724
H	0.86888751325868	-2.65624520888657	-4.48459468909373
H	0.93140538465498	-1.06008442211543	-4.92363479129746
H	-0.53097009329228	0.12102682246283	-2.61720981884681
H	0.99144239533679	0.77104284423388	-3.22866797470319
H	0.59356180585435	1.43355964953941	-0.85205683727437
H	2.06075029219961	0.46676614767992	-1.02019310348542

Section S7. Derived pH Protonation Equation

Equations (S1) and (S2) represent the dissociation equilibria for a diprotic acid, according to the Law of Mass Action. Equation (S3) simply defines the formal concentration for diprotic acids, and Equations (S4)–(S6) give the fractional composition for each protonation state with respect to this formal concentration.

Each α tells you what fraction of the total formal concentration F is in a given protonation state at a certain pH. In our context:

F = the total concentration of your diamine (regardless of whether it's fully protonated, half-protonated, or neutral).

α_{H_2A} = fraction of divalent DACH counterions in the system.

α_{HA^-} = fraction of monovalent DACH counterions in the system.

$\alpha_{A^{2-}}$ = fraction of neutral DACH counterions in the system.

Explanation of How the Equations Are Derived

Equations (S1) and (S2) represent the dissociation equilibria for a diprotic acid according to the Law of Mass Action. Equation (S3) defines the formal concentration of the diprotic acid, and Equations (S4)–(S6) give the fractional composition of each protonation state relative to this formal concentration.

$$K_{a1} = \frac{[HA^-][H^+]}{[H_2A]} \quad (S1)$$

$$K_{a2} = \frac{[A^{2-}][H^+]}{[HA^-]} \quad (S2)$$

$$F = [H_2A] + [HA^-] + [A^{2-}] \quad (S3)$$

$$a_{H_2A} = \frac{[H_2A]}{F} \quad (S4)$$

$$a_{HA^-} = \frac{[HA^-]}{F} \quad (S5)$$

$$a_{A^{2-}} = \frac{[A^{2-}]}{F} \quad (S6)$$

Rearranging Equation (S1) such that $[HA^-]$ is expressed in terms of the other variables yields Equation (S7).

$$[HA^-] = \frac{K_{a1}[H_2A]}{[H^+]} \quad (S7)$$

Rearranging Equation (S2) such that $[A^{2-}]$ is expressed in terms of the other variables yields Equation (S8).

$$[A^{2-}] = \frac{K_{a2}[HA^-]}{[H^+]} \quad (S8)$$

Substituting the definition of $[HA^-]$ given by Equation (S7) into Equation (S8) yields Equation (S9).

$$[A^{2-}] = \frac{K_{a1}K_{a2}[H_2A]}{[H^+]^2} \quad (S9)$$

The definitions of $[HA^-]$ and $[A^{2-}]$ given in Equations (S7) and (S9) can be substituted into Equation (S3) to yield Equation (S10), which gives another expression for the formal concentration.

$$F = [H_2A] + \frac{K_{a1}[H_2A]}{[H^+]} + \frac{K_{a1}K_{a2}[H_2A]}{[H^+]^2} \quad (S10)$$

Factoring $[H_2A]$ from this expression yields a simplified equation in Equation (S11).

$$F = [H_2A](1 + \frac{K_{a1}}{[H^+]} + \frac{K_{a1}K_{a2}}{[H^+]^2}) \quad (S11)$$

This can be further simplified by factoring $1 / [H^+]^2$ from Equation (S11) to yield Equation (S12).

$$F = \frac{[H_2A]}{[H^+]^2}([H^+]^2 + K_{a1}[H^+] + K_{a1}K_{a2}) \quad (S12)$$

Substituting this definition of the formal concentration into Equations (S4)–(S6) yields Equations (S13)–(S15).

$$a_{H_2A} = \frac{[H_2A]}{\frac{[H_2A]}{[H^+]^2}([H^+]^2 + K_{a1}[H^+] + K_{a1}K_{a2})} \quad (S13)$$

$$a_{HA^-} = \frac{K_{a1}[H_2A]}{\frac{[H_2A]}{[H^+]^2}([H^+]^2 + K_{a1}[H^+] + K_{a1}K_{a2})} \quad (S14)$$

$$a_{A^{2-}} = \frac{[A^{2-}]}{\frac{[H_2A]}{[H^+]^2}([H^+]^2 + K_{a1}[H^+] + K_{a1}K_{a2})} \quad (S15)$$

Equations (S14) and (S15) can be modified so that their numerators are given in terms of [H_2A] by substituting Equations (S7) and (S9) into them, respectively. This yields a new set of fractional composition equations:

$$a_{H_2A} = \frac{[H_2A]}{\frac{[H_2A]}{[H^+]^2}([H^+]^2 + K_{a1}[H^+] + K_{a1}K_{a2})} \quad (S13)$$

$$a_{HA^-} = \frac{\frac{K_{a1}[H_2A]}{[H^+]}}{\frac{[H_2A]}{[H^+]^2}([H^+]^2 + K_{a1}[H^+] + K_{a1}K_{a2})} \quad (S16)$$

$$a_{A^{2-}} = \frac{\frac{K_{a1}K_{a2}[H_2A]}{[H^+]^2}}{\frac{[H_2A]}{[H^+]^2}([H^+]^2 + K_{a1}[H^+] + K_{a1}K_{a2})} \quad (S17)$$

Cancelling the common [H^+] and [H_2A] terms in these equations yields:

$$a_{H_2A} = \frac{1}{\frac{1}{[H^+]^2}([H^+]^2 + K_{a1}[H^+] + K_{a1}K_{a2})} \quad (S18)$$

$$a_{HA^-} = \frac{K_{a1}}{\frac{1}{[H^+]}([H^+]^2 + K_{a1}[H^+] + K_{a1}K_{a2})} \quad (S19)$$

$$a_{A^{2-}} = \frac{K_{a1}K_{a2}}{([H^+]^2 + K_{a1}[H^+] + K_{a1}K_{a2})} \quad (S20)$$

Simplifying these equations by removing the fraction in the denominators yields the desired form of the fractional composition equations:

$$a_{H_2A} = \frac{[H^+]^2}{[H^+]^2 + K_{a1}[H^+] + K_{a1}K_{a2}} \quad (S21)$$

$$a_{HA^-} = \frac{K_{a1}[H^+]}{[H^+]^2 + K_{a1}[H^+] + K_{a1}K_{a2}} \quad (S22)$$

$$a_{A^{2-}} = \frac{K_{a1}K_{a2}}{[H^+]^2 + K_{a1}[H^+] + K_{a1}K_{a2}} \quad (S23)$$

The only distinction between Equations (S21)–(S23) and those utilized in the manuscript is the symbolism used to represent each protonation state. Generalized symbols are used in this derivation to provide consistency with the source material, *Quantitative Chemical Analysis (9th ed.)*. In the manuscript, the chosen symbolism reflects the specific protonation states of the diamine counterions.

Assumptions/Limitations

The first major assumption made by this derivation is that there are no other simultaneous processes which could affect the position of equilibrium for the dissociations represented by Equations (S1) and (S2). In other words, reversible processes of micellization and counterion binding are not accounted for when estimating diamine counterion charges. Furthermore, the pre-equilibrium approximation is used, which assumes the first step has achieved thermodynamic equilibrium before the second step begins. This may yield problematic charge estimates for diamine counterions whose dissociations are not significantly different in favorability. The assumption is less certain for counterions where pKa values are relatively close.
